# QuanTest2: benchmarking multiple sequence alignments using secondary structure prediction

**DOI:** 10.1093/bioinformatics/btz552

**Published:** 2019-07-11

**Authors:** Fabian Sievers, Desmond G Higgins

**Affiliations:** Conway Institute, UCD School of Medicine, University College Dublin, Belfield, Dublin 4, Ireland; Conway Institute, UCD School of Medicine, University College Dublin, Belfield, Dublin 4, Ireland

## Abstract

**Motivation:**

Secondary structure prediction accuracy (SSPA) in the QuanTest benchmark can be used to measure accuracy of a multiple sequence alignment. SSPA correlates well with the sum-of-pairs score, if the results are averaged over many alignments but not on an alignment-by-alignment basis. This is due to a sub-optimal selection of reference and non-reference sequences in QuanTest.

**Results:**

We develop an improved strategy for selecting reference and non-reference sequences for a new benchmark, QuanTest2. In QuanTest2, SSPA and SP correlate better on an alignment-by-alignment basis than in QuanTest. Guide-trees for QuanTest2 are more balanced with respect to reference sequences than in QuanTest. QuanTest2 scores correlate well with other well-established benchmarks.

**Availability and implementation:**

QuanTest2 is available at http://bioinf.ucd.ie/quantest2.tar, comprises of reference and non-reference sequence sets and a scoring script.

**Supplementary information:**

Supplementary data are available at *Bioinformatics* online

## 1 Introduction

Multiple sequence alignment (MSA) is an important step in many bioinformatics analyses. The choice of MSA algorithm may be informed by its performance on one or many MSA benchmarks. MSA benchmarks are collections of nucleotide or amino acid sequences, for which the perfect reference alignment is assumed to be known. Construction of protein reference alignments may make use of the proteins’ three-dimensional (3D) structure and/or manual adjustment. It may be difficult to obtain large number of homologous 3D structures, so that the number of reference sequences in benchmark alignments tends to be small. For example, the largest alignment in BAliBASE3 (Thompson *et al.*, 2005) comprises of 142 sequences, with a median of 20 sequences for 218 alignments. The alignment programme is then used to construct an MSA from the unaligned benchmark sequences. The constructed MSAs are compared to the reference alignments and the similarity between the two is expressed in terms of the total column (TC) score or the sum-of-pairs (SP) score (Thompson *et al.*, 1999). The TC score is the fraction of entire alignment columns, shared between reference and test alignment. The SP score is the number of correctly aligned letter pairs divided by the number of aligned pairs in the reference.

The number of available homologous sequences for most protein families is growing rapidly (El-Gebali *et al.*, 2019) and new alignment software is being designed to deal with these increased numbers. One therefore needs large MSA benchmarks to probe the new algorithms’ performance and accuracy.

Simulation can produce reference alignments of virtually any size and with perfectly known phylogeny (Dalquen *et al.*, 2012). However, the alignments strongly depend on the underlying simulation model, which may not always reflect biologically observed reality (Boyce *et al.*, 2015a). In this article, we will look at the relationship between MSA quality and physically observable protein structure and will not consider simulation.

The number of sequences in a benchmark can be increased by mixing reference sequences, for which a reliable structural alignment is known, with homologous non-reference sequences. The earliest example of such a benchmark is PREFAB (Edgar, 2004). It is a collection of 1682 families, where sequence sets are comprised of at most 50 sequences (median 50, mean 45.2). However, every PREFAB sequence sets contains only two reference sequences. All sequences are aligned but only agreement of the alignment of embedded reference sequences can be scored. Another benchmark, based on embedding, is HomFam (Sievers *et al.*, 2011), which comprises of 95 families of up to 93 681 sequences (median 3349), with between 5 and 41 reference sequences (mean 8.3). Each family sequence set is made by combining a small set of sequences from a Homstrad (Mizuguchi *et al.*, 1998) structural alignment with a large number of sequences from Pfam (Finn *et al.*, 2013).

Almost all the contemporary algorithms able to align large number of sequences, employ progressive alignment (PA). In PA, sequences are not all aligned simultaneously but as pairs of individual sequences or smaller alignments to form larger alignments. The order in which these pairwise alignments are performed affects the final alignment and can be encoded as a ‘guide-tree’ (Higgins *et al.*, 1992). The relative alignment of sequences aligned early on in the process can never be changed by subsequent alignments. If the embedded reference sequences are aligned early on, then the quality of the alignment, as expressed by the SP or TC scores of the embedded references, is determined by the alignment of only a few sequences, even if the total number of sequences is large. This is one of the shortcomings of embedded benchmarks like HomFam.

Recently, a new class of benchmark has been explored, which measure the quality of an MSA in terms of the quality of a structure prediction using this MSA. ContTest (Fox *et al.*, 2016) uses the accuracy of a contact map prediction, while QuanTest (Le *et al.*, 2017) uses secondary structure prediction by Jpred4 (Drozdetskiy *et al.*, 2015). In both benchmarks, all sequences in the alignment contribute to the final score, regardless of where the reference sequences are placed in the guide-tree. In ContTest, only one reference sequence was used. For this sequence a real contact map, based on its 3D structure is compared to predicted contacts from an analysis of co-varying residue columns in the alignment (Marks *et al.*, 2011). In QuanTest, the secondary structure prediction accuracy (SSPA) was calculated for three reference sequences; their real secondary structure is compared to the predicted secondary structure of the whole alignment. Using more than one reference in QuanTest allowed us to also calculate SP scores by comparing the structural alignment of the three reference sequences with the test alignment. The final result in Le *et al.* (2017) showed that there was a clear, linear relationship between SP and SSPA score, when different aligners were compared. This was encouraging as it suggested that determining the SP score or the SSPA score both are measuring the same underlying MSA accuracy. However, a comparison of the two benchmarks shows considerable variation and inconsistency on a family-by-family basis (Sievers and Higgins, 2018 and Supplementary Material S1 and S2). In this article, we examine these inconsistencies in detail and create a new version of the benchmark (QuanTest2) which gives more stable and consistent results. This avoids some of the pitfalls of using reference sequences that are too closely related and gives a benchmark that really measures MSA alignment quality on a large scale.

## 2 Approach

The basis of this article is to use the way the SSPA score in QuanTest can be compared to the SP score of the Homstrad sequences embedded in the QuanTest dataset to express the quality of an MSA. When scores for different MSA packages are averaged over all 151 QuanTest families, we see a strong agreement between the two benchmarks (Le *et al.*, 2017).

**Fig. 1.**
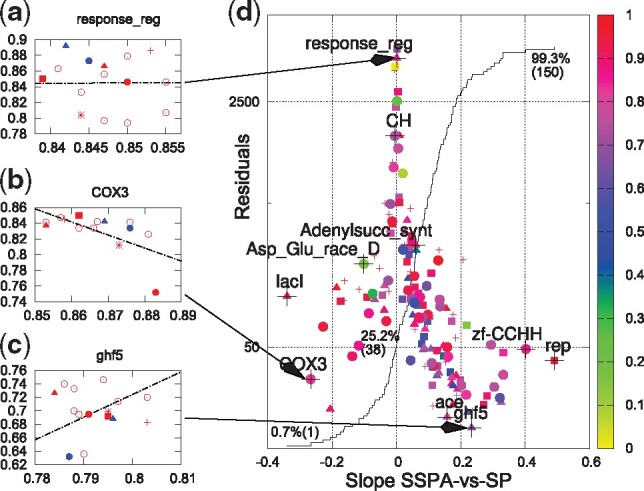
Panels (**a**), (**b**) and (**c**) show SP (along the *y*-axis) against SSPA scores (along the *x*-axis) for response_reg, COX3 and ghf5. Each data point represents one alignment using one aligner/command-line combination only, red points for Clustal Omega, blue for MAFFT options. Regression lines are shown in black. Aligner symbols in (a), (b) and (c) are explained in Sievers and Higgins (2018) and in Supplementary Table S1. Residuals of data points are plotted over slope of regression lines in panel (**d**) for all families. Colour of points in (d) represents the average pairwise identity. Shape of points indicates size of effective guide-tree size: small crosses: 3–400, squares: 401–700, triangles: 701-900, bullets: 901–1000. Large black crosses and labels indicate example families. Faint solid line in (d) is cumulative distribution of slopes, 38 families (∼25%) have negative slope

However, this relationship does not hold on an alignment-by-alignment basis (Sievers and Higgins, 2018). This is demonstrated in Figure 1. There, SP scores for the Homstrad reference sequences as part of the QuanTest dataset are plotted against SSPA scores of the QuanTest framework for three example families, using 14 different alignments made using MAFFT (Katoh and Standley, 2014) and Clustal Omega (Sievers *et al.*, 2011) with a range of alignment options. The exact alignment options are given in Supplementary Table S1. Data points for the protein family response_reg in Figure 1a exhibit no particular correlation, i.e. a linear fit is flat, as calculated by the Levenberg–Marquardt algorithm, implemented in gnuplot (http://www.gnuplot.info). Also, the error, as measured by the residuals, is large, that is, in the top 2% of values encountered. This plot says that there is little or no agreement between the SP and SSPA scores of the QuanTest data for this family, when we use 14 different alignment options. The residuals and regression line slopes are recorded for all 151 protein families in Figure 1d, where the size of the residuals is plotted against the slope of the regression line, and the data point for response_reg is positioned in the top/middle of the panel. Figure 1b shows SP and SSPA scores for 14 alignments of the COX3 data in QuanTest. These points exhibit a strong negative correlation with low residuals, leading to a point in the bottom/left of Figure 1d. This family shows a clear disagreement between the two benchmarking methodologies. The data for family ghf5 in Figure 1c show a positive correlation, represented by a point in the bottom/right of Figure 1d. This family shows clear agreement between the two benchmarking methodologies. There are 38 points (∼25%) indicating negative slopes in Figure 1d.

One problem with directly comparing SP and SSPA scores for the aligners/options in Figure 1 is that they *do not* compare the same alignments. As pointed out in Le *et al.* (2017), SSPA measures the accuracy of the entire alignment. However, due to the nature of PA the SP score measures the quality of the smallest profile that contains all embedded reference sequences. This alignment is the *effective alignment* with respect to the reference sequences and is in general smaller than the total alignment. This effective alignment is produced by the smallest section of the guide-tree that subtends all the embedded reference sequences; this is called the *effective guide-tree* with respect to the embedded reference sequences (Supplementary Material S3). The size of the effective guide-trees is determined by the different aligners/options and can attain arbitrary values. Small effective alignment size, sometimes based on the alignment of just a few sequences, can make SP scores for Homstrad reference alignments embedded in QuanTest erratic.

## 3 Materials and methods

**Fig. 2.**
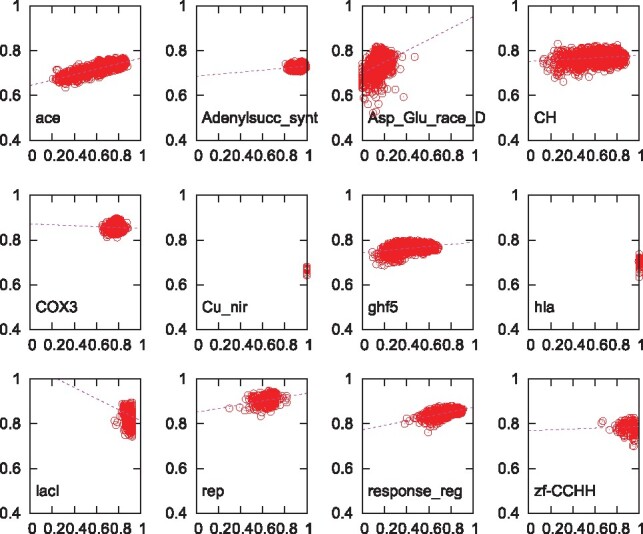
SP (along the *x*-axis) versus SSPA scores (along the *y*-axis) for 12 example families, identified in Figure 1. Each panel contains 1200 points and each point represents one alignment of the original 1000 sequences (Le *et al.*, 2017). Dashed line represents best linear fit

We want to minimize this arbitrariness from the comparison of the SP and SSPA scores. We therefore generate a very large number of external guide-trees (Sievers *et al.*, 2014), where we can guarantee a minimum value of the effective guide-tree size, which we set to 900 (out of 1000 taxa). Basically, we take default guide-trees from a number of different alignment packages and re-root them until the effective guide-tree size with respect to the reference sequences exceeds our minimum threshold of 900 (Felsenstein, 1989). The details of generating these external guide-trees and the aligners/options used to produce the alignments are explained in Supplementary Material S4. The results for 1200 alignments of 12 example families from the original data selection (Le *et al.*, 2017) are shown in Figure 2. The example families are marked up in Figure 1 and were chosen to represent families in different sectors of Figure 1. These are (i) rep, zf-CCHH, ghf5 and ace, which apparently confirm our assumption that SP and SSPA scores should be positively correlated; (ii) lacI, COX3 and Asp_Glu_race_D, which apparently contradict our assumption; (iii) response_reg, CH and Adenylsucc_synt, which are indifferent with large residual errors and (iv) hla and Cu_nir, which were singular and could therefore not be represented in Figure 1. SP scores are calculated using qscore (Edgar, 2004).

Figure 2 replicates the findings in Figure 1, that most, but not all, families exhibit a strong positive correlation between SP and SSPA scores. Since the effective guide-tree size is at least 900 out of the total number of 1000 sequences, we conclude that in QuanTest (Le *et al.*, 2017) the non-reference sequences are not always well matched to the references. This can be seen in Supplementary Material S5. There we show a multi-dimensional scaling of the alignment distances for three example families. In some families the references have a good spread and cover the space of non-reference sequences sufficiently. For other families the reference sequences are clumped in one small section of the alignment space.

We therefore decided to select the sequences for QuanTest2 non-randomly. First, if there are more than three Homstrad sequences available we select reference sequences such that they cover a large volume of the alignment distance space; this is the case for 88 out of the 151 families. Second, we attempt to surround the references by an approximately equal number of non-reference sequences. For this we (i) select sequences from the immediate neighbourhood of the references, (ii) sequences that are close to one reference but far from the other two and (iii) sequences that lie ‘between’ two or three references, as measured by the deviation from the triangle inequality. Of the two steps, the reference selection is the more important. This had been demonstrated in Figure 2 in Le *et al.* (2017). Devising this selection scheme is the main improvement for QuanTest2 over QuanTest.

The aligners used in this study are:

1. Clustal Omega Default (v1.2.3), ––threads=1 -i <in> -o <out> ––guidetree-out=<dnd> (Sievers *et al.*, 2011)
2. Clustal Omega Full, ––threads=1 -i <in> -o <out> ––guidetree-out=<dnd> ––full (Blackshields *et al.*, 2010)
3. Clustal Omega HMM, ––threads=1 -i <in> -o <out> ––guidetree-out=<dnd> ––hmm-in=<hmm>
4. Clustal Omega Iter1, ––threads=1 -i <in> -o <out> ––guidetree-out=<dnd> –iter=1
5. Clustal Omega Iter2, ––threads=1 -i <in> -o <out> ––guidetree-out=<dnd> –iter=2
6. Clustal Omega Viterbi, ––threads=1 -i <in> -o <out> ––guidetree-out=<dnd> –MAC-RAM=1
7. ClustalW2 (v2.1), -INFILE=<in> -OUTFILE=<out> -QUIET -OUTPUT=FASTA -CLUSTERING=UPGMA (Larkin *et al.*, 2007)
8. Decipher (Nov. 2018), R script, generated in time, see Supplementary Material S13 (Wright, 2015)
9. Dialign (v2.2.2), -fa <in> (Morgenstern, 2014)
10. Famsa (v1.2.5), -t 1 -gt_export <dnd> <in> <out> (Deorowicz *et al.*, 2016)
11. FSA (v1.15.9), <in> > <out> (Bradley *et al.*, 2009)
12. Kalign (v2.04), <in> -format fasta -quiet -printtree <dnd> > <out> (Lassmann and Sonnhammer, 2005)
13. Mafft (v7.407), ––anysymbol ––quiet ––thread 1 ––treeout <in> > <out> (Katoh and Standley, 2014)
14. Mafft L-INS-i, ––localpair ––anysymbol ––quiet ––thread 1 –treeout <in> > <out>
15. Mafft PartTree, ––parttree ––anysymbol ––quiet ––thread 1 –treeout <in> > <out>
16. Mafft PartTreeDP, ––dpparttree ––anysymbol ––quiet ––thread 1 ––treeout <in> > <out>
17. Muscle (v3.8.31), -in <in> -out <out> -quiet -tree2 <dnd> (Edgar, 2004)
18. Muscle2, -in <in> -out <out> -quiet -tree2 <dnd> -maxiters 2
19. MSAProbs (v0.9.7), -o <out> -num_threads 1 <in> (Liu and Schmidt, 2014)
20. Opal (v2.1.3), ––mem 10G ––protein ––treeout dnd ––in <in> ––out <out> (Wheeler and Kececioglu, 2007)
21. Pasta (v1.6.3), ––input=<in> ––datatype=Protein ––num-cpus=1 (Mirarab *et al.*, 2015)
22. PastaM, ––input=<in> ––datatype=Protein ––num-cpus=1 ––aligner=muscle
23. PastMM, ––input=<in> ––datatype=Protein ––num-cpus=1 –merger=muscle
24. PastaMMM, ––input=<in> –datatype=Protein ––num-cpus=1 ––aligner=muscle ––merger=muscle
25. POA (V2), -read_fasta <in> -clustal <out> poaV2/blosum80.mat (Lee *et al.*, 2002)
26. Praline (Nov. 2018), ––threads 1 ––output-format fasta ––quiet <in> <out> (Bawono and Heringa, 2014)
27. Prank (v.170427), -d=<in> -o=<out> -f=fasta -seed=1 -quiet -showtree -uselogs (Loytynoja, 2014)
28. Probcons (v1.12), <in> > <out> (Roshan, 2014)
29. TCoffee (v11.00.8cbe486), -in <in> -output fasta -n_core 1 -tree_mode upgma (Magis *et al.*, 2014)
30. UPP (v2.0), -s <in> -m amino ––cpu 1 (Nguyen *et al.*, 2015)

**Fig. 3.**
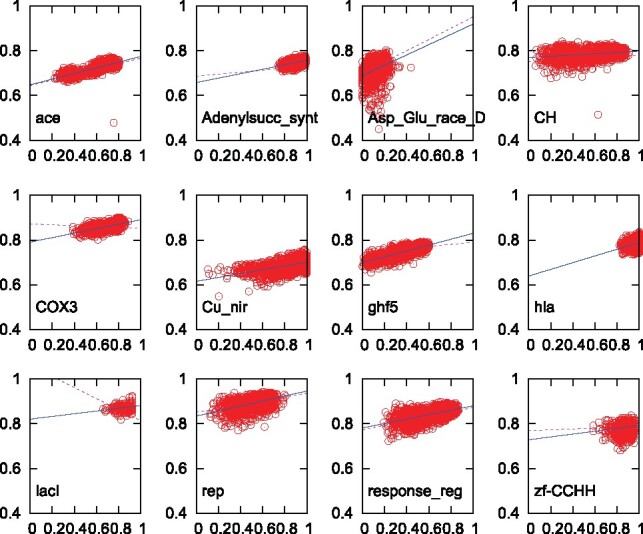
SP versus SSPA scores for 12 example families, identified in Figure 1. Each point represents one alignment of 1000 sequences using the new reference/test selection. Best linear fit for new data selection represented by solid line, best linear fit from old data selection in Figure 2 by dashed line

Using this new selection procedure, we re-generate the 1200 alignments, comprised of 1000 sequences, for each of the 12 example families. The results are shown in Figure 3. One can see that the regression lines for all 12 example families now have positive slope. Specifically, COX3 and lacI are no longer negative, Cu_nir and hla are no longer singular and Adenylsucc_synt, ghf5 and zf-CCHH now exhibit steeper slopes. This can be appreciated by comparing the solid blue regression lines for the new data selection to the broken magenta regression lines for the old data in Figure 2. For over half of the families, the correlation appears to be very tight; this is quantified in Supplementary Table S4. We therefore conclude that the new sequence selection procedure for QuanTest2 predisposes alignments toward strong positive correlation of SP and SSPA scores, provided the effective guide-tree size is closer to the total number of sequences (Supplementary Material S9).

**Fig. 4.**
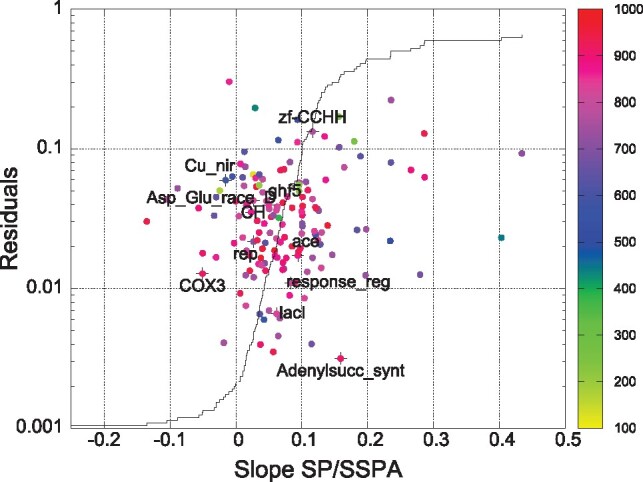
Residuals plotted against slopes of regression lines as in Figure 1. Twelve example families are marked up with crosses and labels. Regression lines are fitted to *N* = 101 alignments, comprised of 1000 sequences. Solid black curve is cumulative distribution of slopes, intersecting *y*-axis at 17 (∼11%). Point colour encodes average effective guide-tree size (yellow/green low, blue medium, magenta/red high)

We now present SP versus SSPA results for a selection of 30 MSA programmes, which are listed above with their respective command lines. However, 30 alignments are still a relatively small number for determining reliable regression lines. One way to increase the number of alignments without increasing the number of aligners/options is to use alignment instability (Boyce *et al.*, 2015b) which comes from different alignments being generated when the sequence input order is changed. We generate new sequence input sets by shuffling the order of sequences in the starting set (see Supplementary Material S8). Depending on the algorithms’ response times, we re-shuffle the full sets of 1000 sequences a number of times and increase thereby the number of alignments to 101. Details of the degree of instability are given in Supplementary Material S8. The results can be seen in Figure 4. There are still families with negative SP versus SSPA slopes, but their number has been reduced from 38 in Figure 1 to 17, and the absolute value of the negative slopes has been reduced. Negative slope values in Figure 4 are not necessarily incompatible with positive slopes in Figure 3 because of statistical fluctuations. This can be seen in Supplementary Figure S9. Results for alignments of 200 sequences of the 151 (new) QuanTest2 families are given in Supplementary Material S6.

While the correlation of the SP and SSPA scores has been improved, the average SP and SSPA scores themselves did not change appreciably; this can be seen in Supplementary Figures S16 and S17. On an average SP scores were decreased, indicating that the original data selection was ‘too easy’, while the average SSPA scores were very slightly increased. The artificial guide-trees used to generate the alignments that were scored in Figures 2 and 3 were constructed such that their effective guide-tree sizes ideally were larger than 900. Guide-tree sizes of alignments represented in Figures 1 and 4 depend on the actual MSA method and are usually smaller than for the artificial guide-trees. These values are compared in Supplementary Tables S7 and S8.

**Fig. 5.**
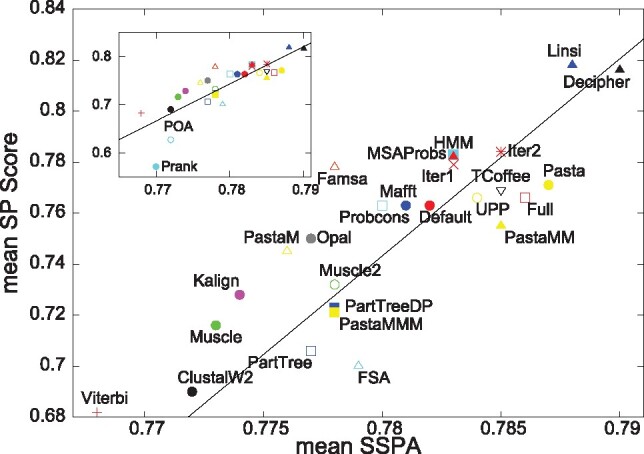
Means of SP versus SSPA scores for different aligners, averaged over all 151 QuanTest2 families and re-samples. Alignments are comprised of 1000 sequences. Solid line is best linear fit of data points. Red symbols for Clustal Omega, dark blue for MAFFT. Inset in top-left corner is overview, capturing outliers, which are omitted in the main panel. Numerical values listed in Table 1

The ultimate purpose of an alignment benchmark is to rank aligners. This can be seen in Figure 5, where we plot the mean SP score over the mean SSPA score for different aligners. Labels are defined above. Results are averaged over 151 QuanTest2 families and the number of re-samples. Numerical values, including resource requirements, are presented in Table 1. Visual inspection suggests that SP and SSPA scores are positively correlated; the Pearson correlation is 0.826 and the Spearman rank correlation is 0.851. MAFFT L-INS-i and Decipher (Wright, 2015) are the most accurate aligners, while Prank (Loytynoja, 2014) and Clustal Omega’s Viterbi mode are the least accurate. Prank’s SP score is substantially worse than any of the other aligners’, however, Prank’s SSPA score does fall within the range of the other aligners. We show the algorithms’ performance on BAliBASE 3.0 (Thompson *et al.*, 2005), PREFAB (Edgar, 2004) and OXBench (Raghava *et al.*, 2003) in Supplementary Material S10.

**Table 1.** Scores and resource consumption for aligners

Aligner/mode	SSPA	SP	*t*	RSS	*r*
Clustal Omega Default	0.782	0.763	100 s	143M	5
Clustal Omega Full	0.786	0.766	98 s	183M	5
Clustal Omega HMM	0.783	0.782	172 s	139M	5
Clustal Omega Iter1	0.783	0.779	292 s	283M	5
Clustal Omega Iter2	0.785	0.784	471 s	283M	5
Clustal Omega Viterbi	0.768	0.682	266 s	566M	5
ClustalW2	0.772	0.690	13 m	*30M*	5
Decipher	**0.790**	*0.816*	100 s	415M	5
Dialign	—	—	65 h	3571M	33/151*
Famsa	0.778	0.778	**5 s**	97M	1^+^
FSA	0.779	0.700	28 h	32G	1
Kalign	0.774	0.728	7 s	**12M**	5
Mafft	0.781	0.763	7 s	177M	5
Mafft L-INS-i	*0.788*	**0.818**	485 s	633M	5
Mafft PartTree	0.777	0.706	*6 s*	303M	5
Mafft PartTreeDP	0.778	0.723	24 s	168M	5
MSAProbs	0.783	0.783	11 h	19G	1
Muscle	0.773	0.716	28 m	385M	5
Muscle2	0.778	0.732	20 s	385M	5
Opal	0.777	0.750	13 m	9G	3
Pasta	0.787	0.771	32 m	169M	2
PastaM	0.776	0.745	24 m	129M	2
PastaMM	0.785	0.755	28 m	126M	2
PastaMMM	0.778	0.721	21 m	130M	2
POA	0.772	0.627	10 m	144M	5
Praline	—	—	258 h	56G	8/151*
Prank	0.770	0.571	19 h	1573M	2
Probcons	0.780	0.763	534 m	15G	1
TCoffee	0.785	0.769	29 h	35G	2
UPP	0.784	0.766	32 m	167M	2

*Note*: Secondary structure prediction accuracy (SSPA), sum-of-pairs (SP) scores and execution time (*t*) in seconds (s), minutes (m) or hours (h) are averaged over all 151 QuanTest2 families and number of re-samples (*r*); Resident set size (RSS) in MB or GB is the maximum value. Best values in bold, second best in italics. Benchmark only partially run for Dialign and Praline (*). FAMSA does not exhibit alignment instability (+). Results produced on a machine with 4 AMD Opteron 6234 processors, 12 cores each, 2 MB cache per core, 2.4 GHz and 256 GB RAM.

## 4 Discussion

Embedded benchmarks are an imperfect solution to the problem of compiling large MSA benchmark sets, using real data. In Figure 3, we demonstrated that if the references are well distributed within the guide tree, then both, SP and SSPA scores are equally well suited to measuring the alignment quality. If, however, the references cluster tightly, then the SSPA is more representative of the entire alignment than the SP score because the latter only assesses the quality of a small part of the alignment. This is the case in Figure 4, where some aligners/options produce guide-trees with a small *effective* size.

We found that both the SP and the SSPA scores for the aligners that we evaluated gave a sensible ordering, which has been suggested by other benchmarks. Decipher and MAFFT L-INS-i were the most accurate, both in terms of SP and SSPA scores. We found that SSPA gave a more benevolent assessment of Prank’s performance, whose gap placement strategy is usually severely penalized by the SP score for non-simulated benchmarks. We also found that Pasta (Mirarab *et al.*, 2015) produces alignments that are only slightly inferior to those produced by MAFFT L-INS-i; this is not surprising, as its subset alignments are constructed using MAFFT L-INS-i. However, if the subset aligner is replaced with MUSCLE, then the quality of the Pasta alignments *exceeds* that of default MUSCLE, suggesting that Pasta’s way of merging profiles (referred to as ‘transitivity’) has a protective effect as the alignment size grows.

The current method can be adapted to select the best alignment of arbitrary sequence sets, if the secondary structure of at least one sequence is reliably known. That is, one can create a plurality of alignments (either by using different aligners, reshuffling the input sequences and/or constructing various external guide trees). One then calculates the SSPA for each of these alignments and selects the alignment which attains the highest SSPA as ‘the best’.

## 5 Conclusion

QuanTest2 is a reconfiguration of the original QuanTest dataset (Le *et al.*, 2017). SP and SSPA scores for MSAs, produced using QuanTest2 correlate positively, when averaged over all families, as well as for (most) individual families. If the correlation is not positive, then the SSPA score should be considered to be more reliable than the SP score for the embedded references as a consequence of the effective guide-tree size. QuanTest2 families are comprised of three Homstrad references and 997 Pfam sequences, which is larger than most available benchmarks. QuanTest2 is available from http://bioinf.ucd.ie/quantest2.tar (Supplementary Material S14).

## Supplementary Material

btz552_Supplementary_Material
