# Supplementary material for: QuanTest2: benchmarking multiple sequence alignments using secondary structure prediction
Source: Bioinformatics. 2019 Jul 11;36(1):90–5. doi: 10.1093/bioinformatics/btz552 (PMC9881607; doi:10.1093/bioinformatics/btz552)
Supplement: btz552_Supplementary_Material [file bioinformatics_36_1_90_s6.pdf]

BIOINFORMATICS

Vol. 00 no. 00 2019
Pages 1–19

# QuanTest2: Benchmarking Multiple Sequence Alignments using Secondary Structure Prediction – Supplemental Material

Fabian Sievers [1,*] and Desmond G Higgins [1*]

[1]School of Medicine and Conway Institute of Biomolecular and Biomedical Research, University College Dublin, Belfield, Dublin 4, Ireland

Received on XXXXX; revised on XXXXX; accepted on XXXXX

Associate Editor: XXXXXXX

**Table S1.** Abbreviations used for aligners (command-lines) in Figures S1 and S3 and Figure 1 in the main paper.

| Abbreviation | Verbose |
|---|---|
| def | Clustal-Ω default |
| full | Clustal-Ω full distance matrix |
| hmm/HMM | Clustal-Ω with Pfam HMM |
| itr1 | Clustal-Ω with one iteration |
| itr2 | Clustal-Ω with two iterations |
| L-INS-i/lnsi | MAFFT L-INS-i (consistency mode) |
| MAFFT/mfft | MAFFT default |
| maxl | Clustal-Ω with ML tree as external guide-tree |
| single/sngl | Clustal-Ω with single linkage tree from full distance matrix |
| t0h1 | Clustal-Ω with no guide-tree and 1 HMM iteration |
| t0h2 | Clustal-Ω with no guide-tree and 2 HMM iterations |
| t1h0 | Clustal-Ω with 1 guide-tree and no HMM iteration |
| t1h2 | Clustal-Ω with 1 guide-tree and 2 HMM iterations |
| t2h0 | Clustal-Ω with 2 guide-tree and no HMM iteration |
| t2h1 | Clustal-Ω with 2 guide-tree and 1 HMM iteration |

'Clustal-Ω' = Clustal Omega

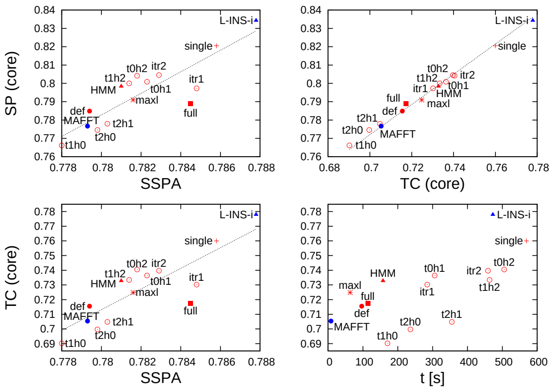


**Fig. S1.** Reproduction of Figure 4 in (Sievers & Higgins, 2018). Only top-left panel, plotting Sum-of-Pairs score against secondary structure prediction accuracy for different aligners (or command-lines) will be considered further. Red symbols are for Clustal Omega command-lines, blue symbols for MAFFT, aligner options are defined in Table S1. Bullets for default mode, triangles for higher-accuracy mode, open circles for iteration modes, cross/star/square for alternative tree building modes.

## S1 INTRODUCTION

Recently we demonstrated how secondary structure prediction can be used to benchmark moderately large to very large alignments (Le *et al.*, 2017). This methodology was used to benchmark various Clustal Omega command-line options and compare them to different other widely used alignment programs (Sievers & Higgins, 2018) and Figure 4 there is reproduced here in Figure S1. Labels identifying the different aligners in this figure and in Figure S2 and Figure S3 are listed in Table S1.

Here we do not focus on run times (bottom-right panel of Figure S1) or in comparing total column (TC) score to Sum-of-Pairs (SP) score (top-right panel). Instead we only want to focus on demonstrating equivalence of the secondary structure prediction accuracy (SSPA) to either the SP or TC score. We arbitrarily choose the SP score (top-left panel) and disregard the TC score (bottom-left panel).

The data that were used to produce the points in Figure S1 are average values for 151 alignments of 1000 protein sequences each. This is illustrated in Figure S2, where the top-left panel of Figure S1 is reproduced in the top-left panel. The other three panels show the results (for three aligners or command-lines) for the individual 151 protein families as small open symbols and the average results as large solid symbols. Three example families are marked up and their relevance will be explained in the next section. The arrows symbolize how the average results for the individual aligners fit into the panel showing results for all aligners or command-lines.

## S2 RESULTS FOR FAMILIES

We now switch our representation from showing results for individual aligners averaged over different protein families to showing results for individual protein families, subsuming different aligners.

To explain this procedure we select three example families: COX3, ghf5 and response_reg. These were the three families that were marked up in Figure S2. We plot SP and SSPA scores for these families in the top-right, bottom-left and bottom-right panels of Figure S3. Using the Levenberg-Marquart algorithm provided by the plotting program `gnuplot` (Williams *et al.*, 2011) we determine regression lines that best fit the scores for the different aligners

*to whom correspondence should be addressed

© Oxford University Press 2019.

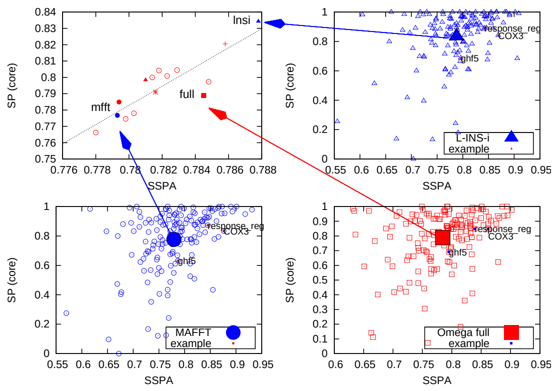


**Fig. S2.** Top-left panel same as top-left panel in Figure S1. Top-right panel shows results for individual protein families as open blue triangles for L-INS-i. Bottom-left panel shows results for individual protein families as open blue circles for default MAFFT. Bottom-right panel shows results for individual protein families as open red squares for full distance matrix Clustal Omega. Average scores are represented as large solid symbols. Average scores in example panels are connected by arrows to their respective positions in the overall panel. In the MAFFT, L-INS-i and Clustal panels three example protein families (COX3, ghf5, response_reg) are marked up; their relevance is explained in the next section.

or command-lines. In the top-left panel of Figure S3 we plot the residuals of the data points with respect to the regression lines over the slopes of the regression lines. The y-axis is logarithmic.

The COX3 data point is in the bottom-left corner, because the regression line in the bottom-left panel of Figure S3 has negative slope and the individual data points are clustered tightly around the regression line. The response_reg data point in the top-left panel of Figure S3 is in the top-centre of the panel because the regression line in the top-right panel of Figure S3 has zero slope and the individual data points are scattered widely about the regression line. The ghf5 data point in the bottom-right panel of Figure S3 is in the bottom-right corner because the regression line in the bottom-right panel of Figure S3 has a positive slope and the individual data points are clustered tightly around the regression line. The ghf5 data confirms our observation, that the SP score correlates (positively) with the SSPA score. The former two *do not* confirm this expectation.

In Figure S4 we show residuals over slopes for all 151 protein families, where two families (hla and Cu_nir) are off-scale and are displayed in the inset in relation to the other families.

We wish to demonstrate that the SSPA score positively correlates with the more traditional SP score and that this correlation is tight. Ideally we would like to see all data points in the bottom-right corner of Figure S4, where the slopes are positive and large, and where the residuals are small. Clearly this is not the case. While there is an appreciable number of points along the 'agree' arrow, there are points that represent anti-correlation (disagree) or show no clear signal (indifferent).

There are some possible explanations for these discrepancies.

1. SP and/or SSPA score are used incorrectly
2. There were not enough points to construct a meaningful regression line
3. The test/reference data were not curated in a representative way

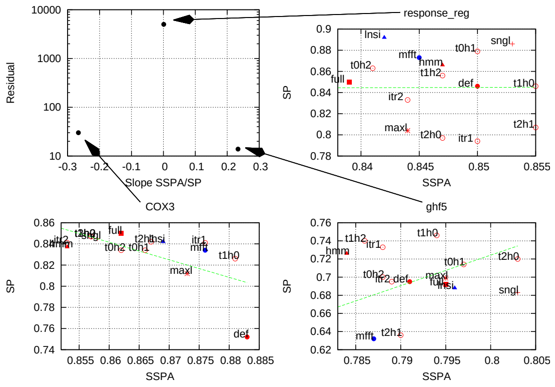


**Fig. S3.** Top-right, bottom-left and bottom-right panels show SP over SSPA scores for response_reg, COX3 and ghf5. Each data point represents one alignment using one particular aligner (or command-line) only. Symbol colour and shape identifying aligner (or command-line) same as in Figure S1. Regression lines in green. Residuals of data points are plotted over slope of regression lines in top-left panel.

## S3 EFFECTIVE TREE SIZE

Like HomFam (Sievers *et al.*, 2011) QuanTest (Le *et al.*, 2017) is an embedded benchmark. A small number of reference sequences, for which their alignment and their secondary structure is known, are embedded in a much larger set of homologous sequences, for which no reliable alignment and/or secondary structure is known. In QuanTest as well as in HomFam the reference sequences are sourced from Homstrad (Mizuguchi *et al.*, 1998), while the majority of non-reference sequences come from Pfam (Finn *et al.*, 2013).

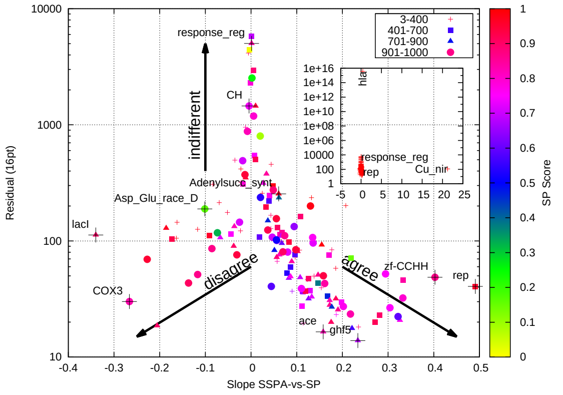


**Fig. S4.** Residuals of data points over slope of regression lines for all 151 protein families. Symbol shape and size represents size of effective guide-tree. Symbol colour represents average Q-score (yellow/green low score, blue ∼0.5, magenta/red high score). Inset shows two off-scale data points in relation to body of data points.

**Table S2.** Aligners used in this study

| | Aligner | Version | Mode | Command-line |
|---|---|---|---|---|
| 1. | Clustal Omega | 1.2.3 | Default | `--threads=1 -i <in> -o <out> --guidetree-out=dnd` |
| 2. | | | Full | `--threads=1 -i <in> -o <out> --guidetree-out=dnd --full` |
| 3. | | | HMM | `--threads=1 -i <in> -o <out> --hmm-in=hmm` |
| 4. | | | Iter1 | `--threads=1 -i <in> -o <out> --guidetree-out=dnd --iter=1` |
| 5. | | | Iter2 | `--threads=1 -i <in> -o <out> --guidetree-out=dnd --iter=2` |
| 6. | | | Viterbi | `--threads=1 -i <in> -o <out> --MAC-RAM=1` |
| 7. | ClustalW2 | 2.1 | ClustalW2 | `-INFILE=<in> -OUTFILE=<out> -QUIET -OUTPUT=FASTA -CLUSTERING=UPGMA` |
| 8. | Decipher | Nov 2018 | Decipher | R script, generated in time, see Supplement S10 |
| 9. | DIALIGN | 2.2.2 | Dialign | `-fa <in>` |
| 10. | Famsa | 1.2.5 | Famsa | `-t 1 -gt_export dnd <in> <out>` |
| 11. | FSA | 1.15.9 | FSA | `<in> > <out>` |
| 12. | Kalign | 2.04 | Kalign | `<in> -format fasta -quiet -printtree dnd > <out>` |
| 13. | MAFFT | 7.407 | Mafft | `--anysymbol --quiet --thread 1 --treeout <in> > <out>` |
| 14. | | | Linsi | `--localpair --anysymbol --quiet --thread 1 <in> > <out>` |
| 15. | | | PartTree | `--parttree --anysymbol --quiet --thread 1 <in> > <out>` |
| 16. | | | PartTreeDP | `--dpparttree --anysymbol --quiet --thread 1 <in> > <out>` |
| 17. | MUSCLE | v3.8.31 | Muscle | `-in <in> -out <out> -quiet -tree2 dnd` |
| 18. | | | Muscle2 | `-in <in> -out <out> -quiet -tree2 dnd -maxiters 2` |
| 19. | MSAProbs | 0.9.7 | MSAProbs | `-o <out> -num_threads 1 <in>` |
| 20. | Opal | 2.1.3 | Opal | `--mem 10G --protein --treeout dnd --in <in> --out <out>` |
| 21. | Pasta | v1.6.3 | Pasta | `--input=<in> --datatype=Protein --num-cpus=1` |
| 22. | | | PastaM | `-i <in> -d Protein --num-cpus=1 --aligner=muscle` |
| 23. | | | PastaMM | `-i <in> -d Protein --num-cpus=1 --merger=muscle` |
| 24. | | | PastaMMM | `-i <in> -d Protein --num-cpus=1 --aligner=muscle --merger=muscle` |
| 25. | POA | V2 | POA | `-read_fasta <in> -clustal <out> poaV2/blosum80.mat` |
| 26. | Praline | Nov 2018 | Praline | `--threads 1 --output-format fasta --quiet <in> <out>` |
| 27. | Prank | v.170427 | Prank | `-d=<in> -o=<out> -f=fasta -seed=1 -quiet -showtree -uselogs` |
| 28. | Probcons | 1.12 | Probcons | `<in> > <out>` |
| 29. | TCoffee | 11.00.8cbe486 | TCoffee | `-in <in> -output fasta -n_core 1 -tree_mode upgma` |
| 30. | UPP | 2.0 | UPP | `-s <in> -m amino --cpu 1` |

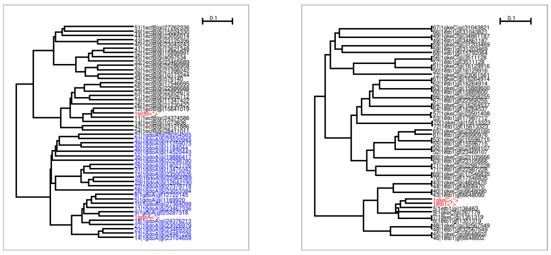

**Fig. S5.** Clustal Omega default guide-trees for two Prefab data sets of 50 sequences. Two reference sequences in red, non-reference sequences in blue or black.

It is one of the shortcomings of embedded benchmarks that they can only assess the TC/SP score for the small number of embedded reference sequences. The alignment of the reference sequences very much depends on the shape of the guide-tree. If all the reference sequences are aligned early on in the progressive alignment scheme, then none of the non-reference sequences that are aligned onto the reference sequences will affect the final TC/SP score – the final TC/SP score is therefore a function of the sequences that are aligned until all reference sequences are part of one profile. The number of sequences in this profile is the 'effective' profile size (with respect to the reference sequences), and the smallest possible sub-tree that subtends all reference sequences is the effective guide-tree (with respect to the reference sequences). This is visualised in Figure S5, using Clustal Omega default (Sievers *et al.*, 2011) alignments of two Prefab (Edgar, 2004) data sets. There, two reference sequences in red are embedded within 48 non-reference sequences in either blue or black. In the left panel all the non-reference sequences in black are first aligned to reference sequence 1ecfB and all the non-reference sequences in blue are first aligned to reference sequence 1gdoA, before the two profiles containing the reference sequences are aligned themselves. In this case the effective tree-size is the full 50 sequences. In the right panel the two reference sequences in red (1gkeC and 1etb1) are immediately aligned to each other, all the non-reference sequences in black are aligned to the reference sequences after this initial alignment. In this case the effective tree-size is two.

However, it is well known (Sievers *et al.*, 2013) that the size of an alignment does influence the quality of this alignment. For small numbers of sequences this quality *may* increase, however, for large numbers the quality inevitably *will* decrease. Structure prediction benchmarks, like ContTest (Fox *et al.*, 2015) or QuanTest on the other hand always assess the quality of the entire alignment. In Figure S3 we may therefore have compared the SSPA score of an alignment of 1000 sequences to the SP score of an alignment of effectively only a fraction of 1000 sequences. Consequently, the slopes and residuals in Figure S4 may be unreliable. This is symbolized by shape and size of the data points. Large bullets correspond to effective sizes of the guide-tree of on average over 900. Medium triangles or squares represent effective guide-tree sizes of on average 700-900 or 400-700, respectively. Small crosses stand for effective sizes of the guide-tree of on average fewer than 400.

The distribution of effective tree sizes for all 151 families in Figure S4 is represented in Figure S6. The distribution of effective tree sizes is rendered with candlesticks, where the whiskers represent the top/bottom quartiles, the boxes the middle quartiles and the bullets the medians. Families are arranged along the x-axis with increasing median effective guide-tree size. 40 families have median effective tree sizes of less than 400 taxa, 74 (=40+34) families have medians of less than 700 taxa and 98 (74+24) families have medians of less than 900 taxa. There are seven families where all effective trees have more than 900 taxa.

The last candlestick, labeled 'Summ', gives the distribution for all (2114=14 alignments$\times$151 families) trees for all families. The overall median effective trees size is 776 taxa.

In Table S3 the effective tree size distribution in Figure S6 is shown and compared to the effective size distribution of the artificial guide-trees for the twelve example families in Figure 2 in the main paper (S4).

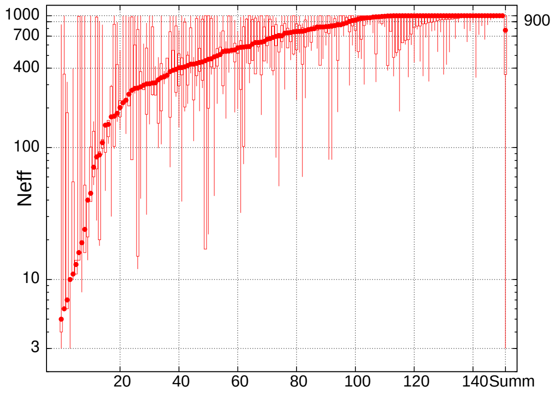


**Fig. S6.** Effective tree size distribution for 151 families in Figure S4. Top/bottom quartiles as whiskers, middle quartiles as box, median as bullet. Candlesticks are sorted according to median. Last candlestick (Summ) is distribution for all effective trees.

**Table S3.** Quartiles of the effective tree sizes for the real trees of the old data selection for 1,000 sequences

| Q0 | Q1 | Q2 | Q3 | Q4 | family |
|---|---|---|---|---|---|
| 435 | 592 | 663 | 1000 | 1000 | ace |
| 595 | 836 | 919 | 1000 | 1000 | Adenylsucc_synt |
| 788 | 834 | 1000 | 1000 | 1000 | Asp_Glu_race_D |
| 425 | 939 | 1000 | 1000 | 1000 | CH |
| 317 | 889 | 1000 | 1000 | 1000 | COX3 |
| 250 | 250 | 310 | 321 | 414 | Cu_nir |
| 595 | 748 | 827 | 841 | 1000 | ghf5 |
| 166 | 536 | 537 | 565 | 640 | hla |
| 470 | 663 | 953 | 1000 | 1000 | lacI |
| 177 | 356 | 630 | 998 | 1000 | rep |
| 296 | 755 | 906 | 1000 | 1000 | response_reg |
| 339 | 998 | 1000 | 1000 | 1000 | zf-CCHH |

**Table S4.** Quartiles of the effective tree sizes for the artificial trees of the old data selection for 1,000 sequences

| Q0 | Q1 | Q2 | Q3 | Q4 | family |
|---|---|---|---|---|---|
| 916 | 943 | 975 | 997 | 1000 | ace |
| 908 | 944 | 975 | 991 | 1000 | Adenylsucc_synt |
| 909 | 958 | 979 | 996 | 1000 | Asp_Glu_race_D |
| 906 | 944 | 978 | 1000 | 1000 | CH |
| 904 | 960 | 978 | 996 | 1000 | COX3 |
| 900 | 962 | 984 | 997 | 1000 | Cu_nir |
| 902 | 924 | 959 | 985 | 1000 | ghf5 |
| 906 | 954 | 984 | 995 | 1000 | hla |
| 910 | 932 | 968 | 998 | 1000 | lacI |
| 900 | 960 | 977 | 1000 | 1000 | rep |
| 902 | 944 | 969 | 989 | 1000 | response_reg |
| 906 | 956 | 985 | 998 | 1000 | zf-CCHH |

## S4 SAMPLE SIZE

In a next step we want to increase the number of data-points that are used to calculate the regression line. It would be prohibitive to dramatically increase the number of alignments for each of the 151 protein families. We therefore select a small but hopefully representative sample of families:

- 4 families that (so far) confirm our hypothesis, that SP score correlates with SSPA score
  - rep
  - zf-CCHH
  - ghf5
  - ace
- 3 families that (so far) clearly contradict our hypothesis
  - lacI
  - COX3
  - Asp_Glu_race_D
- 3 'indifferent families'
  - response_reg
  - CH
  - Adenylsucc_synt
- and 2 off-scale families
  - hla
  - Cu_nir

For these 12 families we generate 1,200 alignments each and calculate their respective SP and SSPA scores. The 1,200 alignments are produced in the following way:

- ($\alpha$.i)we generate 8 initial guide-trees and alignments
  - ($\alpha$.i.1)Clustal Omega default (Sievers *et al.*, 2011)
  - ($\alpha$.i.2)Clustal Omega full distance matrix (Blackshields *et al.*, 2010)
  - ($\alpha$.i.3)Clustal Omega iterated once
  - ($\alpha$.i.4)MAFFT L-INS-i (Katoh & Standley, 2013)
  - ($\alpha$.i.5)MAFFT default
  - ($\alpha$.i.6)MUSCLE default (Edgar, 2004)
  - ($\alpha$.i.7)ClustalW2 neighbour joining (Larkin *et al.*, 2007)
  - ($\alpha$.i.8)ClustalW2 UPGMA
- ($\alpha$.ii)for the above 8 alignments we estimate 8 maximum likelihood trees (Price *et al.*, 2010)

- ($\alpha$.iii)based on the Clustal Omega full distance matrix we construct the following trees (Pavlopoulos *et al.*, 2010)
  - ($\alpha$.iii.1)complete linkage
  - ($\alpha$.iii.2)mean linkage
  - ($\alpha$.iii.3)single linkage
  - ($\alpha$.iii.4)Ward linkage
- ($\beta$.i)Each of the above 20 initial trees is re-rooted (Felsenstein, 1989) several times, such that each initial tree produces 5 trees, where the effective guide-tree size with respect to the reference sequences is at least 900.
- This produces 100 trees.
  - Ideally the initial tree is retained, however, if the effective size of the guide-tree is smaller than 900, then it may be discarded.
- ($\beta$.ii)Varying proportions (10%, 20%, 50%, 80%) of non-reference nodes are randomly swapped.
- This produces another 100 trees.
  - By not swapping reference sequences it is guaranteed that the effective tree-size is at least 900.
- ($\gamma$)Each of the 200 guide-trees is fed into 6 aligners (or command-lines)
  - ($\gamma$.1)Clustal Omega default
  - ($\gamma$.2)Clustal Omega with Pfam HMM
  - ($\gamma$.3)MAFFT L-INS-i
  - ($\gamma$.4)MAFFT default
  - ($\gamma$.5)MUSCLE default
  - ($\gamma$.6)ClustalW2

This produces 1,200 alignments (= 200 guide-trees $\times$ 6 aligners). SP and SSPA for these 1,200 alignments are shown in Figure S7.

It is clear that some families now show a stronger correlation of SP and SSPA scores than before. These are:

- Adenylsucc_synt (caveat – SP range very small)
- CH
- COX3
- Cu_nir (slope is no longer singular)
- hla (residuals are tighter)
- response_reg

zf-CCHH appears to have deteriorated, Asp_Glu_race_D is not particularly tight and lacI is still the family with the strongest anti-correlation. This can be seen in Figure S8. The residuals are calculated for a different number of data points than for Figure S4 so that the normalisation of the y-axis is different.

The regression lines for the 1200 artificial guide-tree date points in Figure 3 of the main paper all have positive slopes. However,

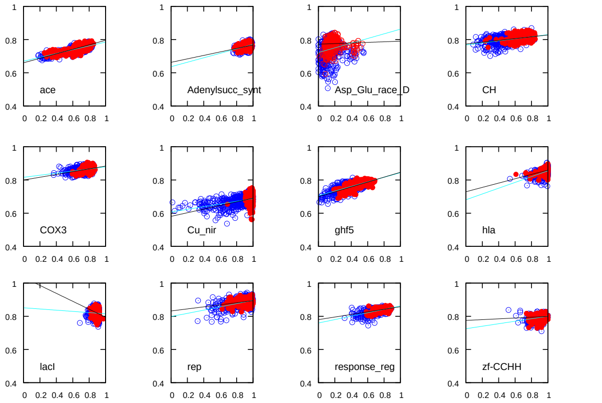


**Fig. S7.** SP over SSPA scores for 12 example families. There are 600 red data points and black regression lines for original ($\beta$.i) trees and 600 blue points and blue regression lines for mutated trees ($\beta$.ii).

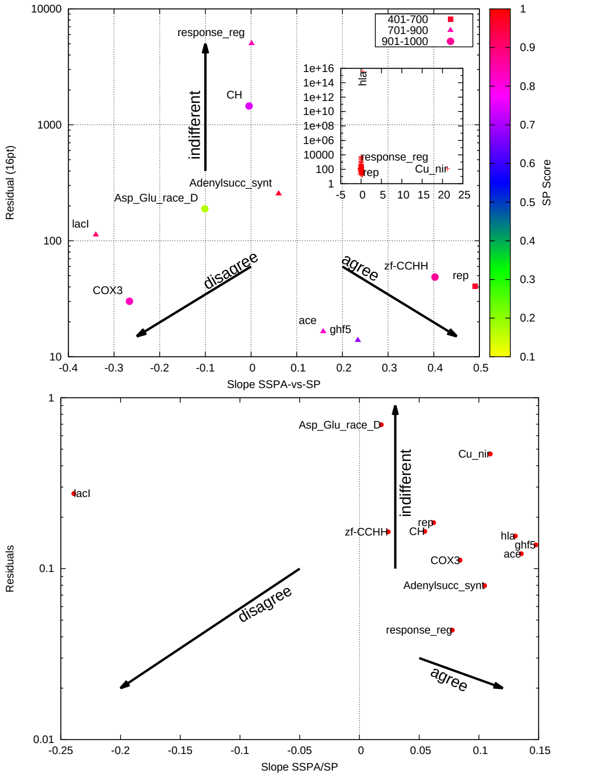


**Fig. S8.** Residuals of data points over slope of regression lines for 12 example families. Top panel same as Figure S4 for 14 alignments, with example families projected out. Slopes/residuals in bottom panel based on 1,200 alignments in Figure S7.

some of the data points in Figure 4 of the main paper indicate negative slopes. This can partially be explained by the fact that the effective guide-tree sizes for the artificial guide-trees are all larger than 900, while the default guide-trees can attain arbitrary values. Another reason is the sample size. We superimposed the 1200 artificial data points and the 101 default tree data points for COX3, which is part of the original example families. We also

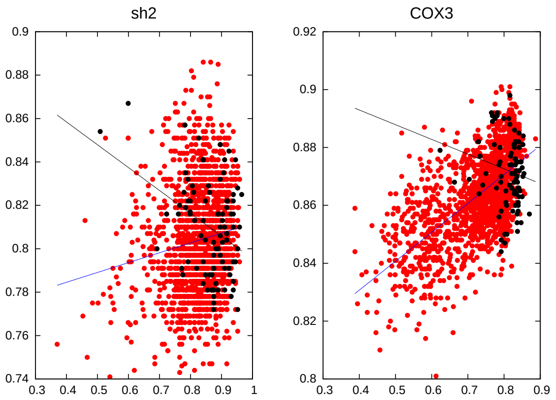


**Fig. S9.** SSPA scores over SP scores for two families, sh2 left, COX3 right. Red points indicate alignments using artificial guide-trees with $N_{eff} \geq 900$, black point for alignments from default guide-trees, that gave rise to points in Figure 4 in the main paper. Regression line for 1200 artificial guide-tree alignments in blue, regression line for 101 default guide-tree alignments in black.

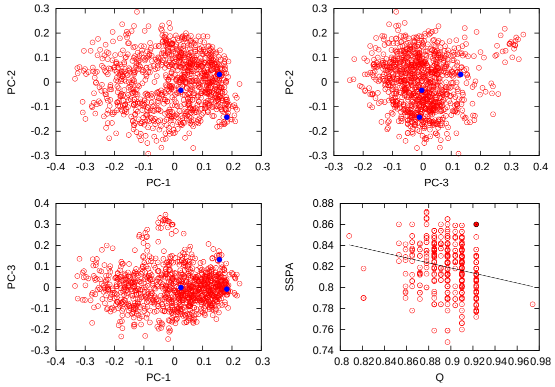


**Fig. S10.** Top-left, bottom-left and top-right panels show embeddings of lacI sequences into first three principal components, accounting for 8.3%, 5.9% and 4.5% of the variation. Reference sequences in blue, non-reference sequences in red. There are 1000 data points representing 1000 sequences. Bottom-right panel shows SP/SSPA scores for 1,200 alignments. Black line is best fit straight line. Red bullet with black rim represents alignment that was used to determine alignment distances used for embedding.

performed a new analysis for sh2, which in Figure 4 attains a large negative value. For both families one can see that the smaller set of data points falls within the cluster of the larger data set. This can be seen in Figure S9. The larger data sets have regression lines with positive slopes, while the smaller data sets have regression lines with negative slopes. This discrepancy can partially be explained by statistical fluctuations.

## S5 DATA SELECTION

So far we have explained (i) how the effective size of the guide-tree can obscure the SP/TC score and (ii) how increasing the number of alignments can improve the SP/SSPA correlation signal. We will now show (iii) how improper sequence selection can lead to poor correlation.

The family that exhibits the worst correlation in Figure S7 is lacI. We hypothesize that a possible reason for this behaviour is that the reference sequences are not well matched to the non-reference sequences (or vice versa). To explore this we try to visualise the relationship of the reference sequences amongst the non-reference sequences.

One way is to calculate distances amongst the sequences and embed the sequences in a low-dimensional space and to decide by visual inspection if the reference sequences are representative of the overall collection of sequences. We used the alignment distances of the best available alignment, which is the one that attains the highest combination of SP and SSPA score.

This can be seen in Figures S10-S12, where we show results for lacI, COX3 and zf-CCHH. The bottom-right panels show the SP/SSPA scores of the original 1,200 alignments that were shown in red in the respective panels in Figure S7. The black lines are the best fit straight lines through the data points. A line with positive slope indicates a positive correlation, a line with negative slope an anti-correlation. The alignment that was considered to be the best in terms of SP and SSPA score is represented by a red bullet with a black rim. This 'best' alignment was used to generate a

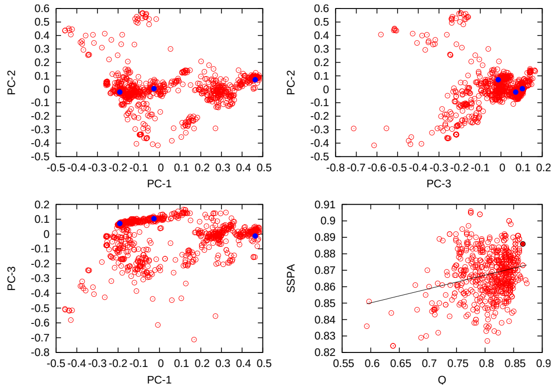


**Fig. S11.** Top-left, bottom-left and top-right panels show embeddings of COX3 sequences into first three principal components. Reference sequences in blue, non-reference sequences in red. There are 1000 data points representing 1000 sequences. Bottom-right panel shows SP/SSPA scores for 1,200 alignments. Black line is best fit straight line. Red bullet with black rim represents alignment that was used to determine alignment distances used for embedding.

matrix of alignment distances which were then embedded in a lower dimensional space. Combinations of the first three dimensions are shown in the top-left, bottom-left and top-right panels. These panels contain 1000 points representing the 1000 sequences. Reference sequences are rendered as blue bullets and non-reference sequences as red circles.

For the lacI data set in Figure S10 the first three dimensions account for 8.3%, 5.9% and 4.5% of the variation, respectively. Visual inspection suggests that the reference sequences are not evenly spread amongst the entire sequence collection. This may account for the poor correlation of the SP and SSPA scores for lacI.

The slope of the regression line for SP/SSPA data points of COX3 in Figure S11 is positive. The first two dimensions of the alignment distance embedding account for 27.6% and 18.3% of the variation.

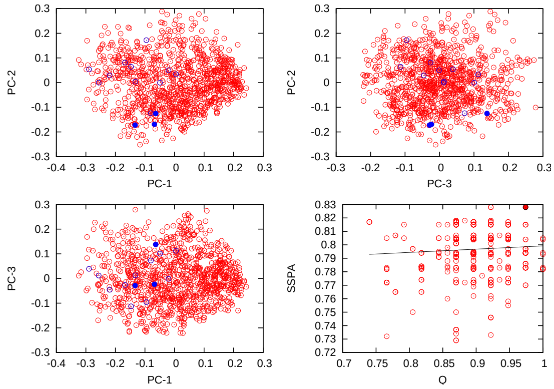


**Fig. S12.** Top-left, bottom-left and top-right panels show embeddings of zf-CCHH sequences into first three principal components. Sequences available as references as blue circles, sequences actually used as references as blue bullets, non-reference sequences in red. There are 1000 data points representing 1000 sequences. Bottom-right panel shows SP/SSPA scores for 1,200 alignments. Black line is best fit straight line.

**Table S5.** Pearson Correlation and Spearman Rank Correlation of SP and SSPA scores for 1,200 alignments of old data selection for twelve example families

| Pearson | Spearman | Family |
|---|---|---|
| 0.83894 | 0.776077 | ace |
| 0.227213 | 0.222152 | Adenylsucc_synt |
| 0.416094 | 0.374418 | Asp_Glu_race_D |
| 0.196872 | 0.105506 | CH |
| -0.0565914 | -0.186619 | COX3 |
| — | — | Cu_nir |
| 0.360547 | 0.269289 | ghf5 |
| 0.0300063 | 0.0409498 | hla |
| -0.173537 | -0.173225 | lacI |
| 0.289925 | 0.265146 | rep |
| 0.659558 | 0.553726 | response_reg |
| 0.0863881 | 0.100075 | zf-CCHH |

However, the third eigenvector is negative (7.6% of variation), suggesting that either the alignment and/or the distances amongst the sequences are problematic.

In Figure S12 we represent the pool of possible reference sequences by blue circles. The sequences that were actually chosen as references are represented by blue bullets. Visual inspection, especially of the top-left panel, showing the first two principal components, suggests that the selection of reference sequences may have been sub-optimal.

SP-vs-SSPA values for the final selection of the twelve example families are presented in Figure 3 of the original paper. There the number of alignments has been increased to 1,800, that is, 600 original guide-trees plus 1,200 mutated guide-trees. The Pearson and Spearman rank correlation coefficients are given in Table S7.

## S6 RESULTS FOR 200 SEQUENCES

To explore the resource requirements of the aligners/options listed in Table S2 we performed an initial alignment run with 200 sequences. Results were produced on a machine with 4 AMD Opteron 6234 processors, 12 cores each (48 cores in total), 2MB cache per core and 256GB RAM. Average SP score, average SSPA score, average run-times as well as the maximum amount of RAM required are given in Table S6. There, one can see that Decipher (Wright, 2015) is the most accurate algorithm, both in terms of SP as well as SSPA scores. MAFFT L-INS-i and UPP Nguyen *et al.* (2015) come in second, in terms of SP and SSPA scores, respectively. Kalign (Lassmann & Sonnhammer, 2005), for 200 sequences, is the fastest algorithm requiring the least amount of memory. A graphic representation of SSPA scores over run-time can be found in Figure S13. The x-axis, showing time, has logarithmic scale, the y-axis for the SSPA score is linear. If time is not an issue, then one should simply choose the best performing program, which in this case is Decipher. However, if the analysis is to be performed repeatedly and timing is important, then the representation in Figure S13 is well suited to select a less accurate but faster compromise algorithm.

For 200 sequences Kalign is the fastest algorithm. However, the Kalign algorithm has quadratic complexity. This is less favourable, than, for example, Famsa or MAFFT PartTree. Therefore Kalign for 1,000 sequences is still very fast but no longer the fastest program.

**Table S6.** Scores and Resource Consumption for Aligners for 200 sequences: Secondary Structure Prediction Accuracy (SSPA), Sum-of-Pairs (SP) scores and execution Time (t) in seconds (s), minutes (m) or hours (h) are averaged over all 151 QuanTest2 families and number of re-samples (r); Resident Set Size (RSS) in MB or GB is the maximum value.

| Aligner/Mode | SSPA | SP | t | RSS | r |
|---|---|---|---|---|---|
| Clustal Omega Default | 0.777 | 0.795 | 10s | 83M | 1 |
| Clustal Omega Full | 0.779 | 0.791 | 12s | 74M | 1 |
| Clustal Omega HMM | 0.781 | 0.807 | 19s | 85M | 1 |
| Clustal Omega Iter1 | 0.780 | 0.805 | 37s | 127M | 1 |
| Clustal Omega Iter2 | 0.780 | 0.805 | 63s | 126M | 1 |
| Clustal Omega Viterbi | 0.772 | 0.751 | 11s | 22M | 1 |
| ClustalW2 | 0.769 | 0.719 | 34s | **5M** | 1 |
| Decipher | **0.783** | **0.823** | 21s | 331M | 1 |
| Dialign | 0.775 | 0.720 | 384s | 171M | 1 |
| Famsa | 0.777 | 0.787 | 1s | 34M | 1 |
| FSA | 0.777 | 0.704 | 65m | 1371M | 1 |
| Kalign | 0.771 | 0.741 | **0.6s** | **5M** | 1 |
| Mafft Linsi | 0.780 | 0.817 | 20s | 75M | 1 |
| Mafft | 0.777 | 0.783 | 1s | 45M | 1 |
| Mafft PartTree | 0.773 | 0.752 | 1s | 37M | 1 |
| Mafft PartTreeDP | 0.779 | 0.764 | 4s | 25M | 1 |
| MSAProbs | 0.779 | 0.779 | 14m | 784M | 1 |
| Muscle | 0.772 | 0.772 | 36s | 80M | 1 |
| Muscle2 | 0.776 | 0.771 | 2s | 80M | 1 |
| Opal | 0.777 | 0.789 | 66s | 3711M | 1 |
| Pasta | 0.779 | 0.809 | 276s | 93M | 1 |
| PastaM | 0.774 | 0.777 | 223s | 86M | 1 |
| PastaMM | 0.780 | 0.795 | 244s | 62M | 1 |
| PastaMMM | 0.775 | 0.763 | 194s | 44M | 1 |
| POA | 0.768 | 0.626 | 34s | 42M | 1 |
| Praline | 0.758 | 0.689 | 29m | 2712M | 1 |
| Prank | 0.771 | 0.630 | 164m | 108M | 1 |
| Probcons | 0.777 | 0.776 | 12m | 637M | 1 |
| TCoffee | 0.780 | 0.793 | 39m | 1407M | 1 |
| UPP | 0.781 | 0.815 | 280s | 92M | 1 |

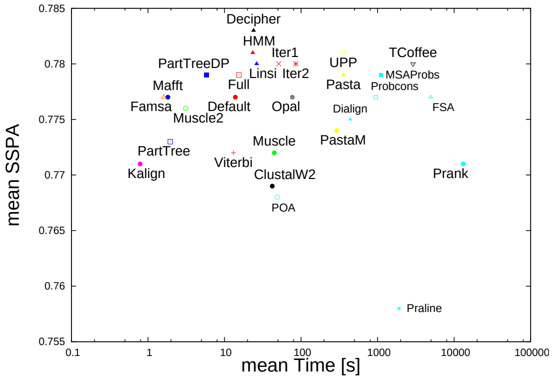


**Fig. S13.** Mean SSPA score versus mean execution time for different algorithms, aligning 200 sequences. Sequences are sampled only once. The x-axis (time) has logarithmic scale, the y-axis (SSPA) is linear. Symbol colours and shapes for different aligners are the same as in Supplemental Figure S15 or main text Figure 5.

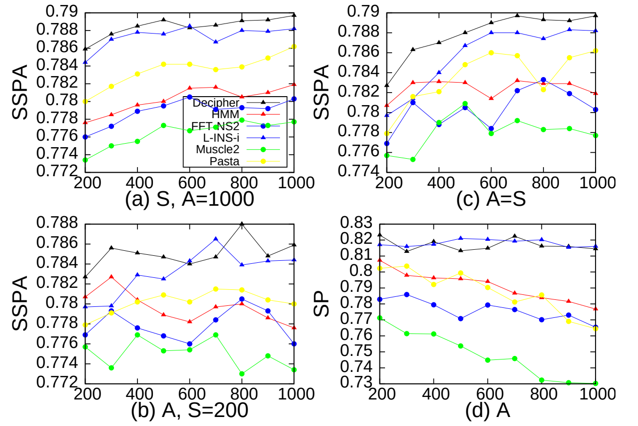


**Fig. S14.** Alignment accuracy for Decipher (black), Clustal Omega HMM (red), MAFFT L-INS-i (blue triangles), default MAFFT (blue bullets), Muscle 2 iterations (green) and Pasta (yellow), as number of sequences is varied. In panel (a) the alignment size is fixed to A=1,000 and the number S of sequences used to generate the SSPA is varied. In panel (b) the alignment size A is varied but only the same S=200 sequences are used to generate the SSPA. In panel (c) all sequences that are aligned are used to generate the SSPA (A=S). Panels (a)-(c) show the SSPA score, panel (d) shows the SP score as the number of sequences A is varied – no S in this case.

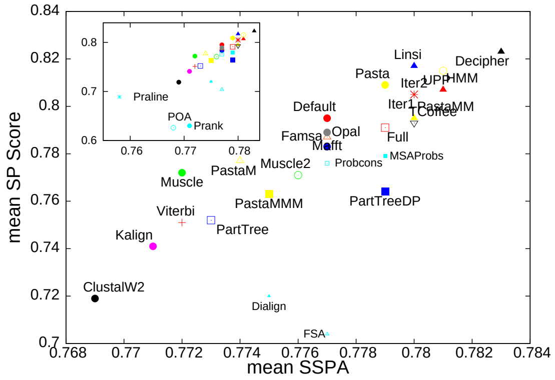


**Fig. S15.** Mean SP scores versus mean SSPA scores for one re-sample of 200 sequences. Aligner labels are identified in Table S2, same as in main paper.

It is well known that for most aligners the alignment quality decreases as the number of sequences is increased to very large numbers (Sievers *et al.*, 2013), however, structure prediction accuracy usually improves with sequence number. This can be seen in Figure S14, where we show results for six aligners and one re-shuffling of the 151 families in QuanTest2. In panel S14(a) we use increasingly larger numbers of sequences from a fixed alignment of 1,000 sequences. The prediction accuracy for all aligners increases with increasing number of aligned sequences, sampled from a fixed alignment. In panel S14(b) we use the same 200 sequences from increasingly larger alignments. It can be seen that for Decipher (Wright, 2015) and MAFFT L-INS-i the SSPA initially increases and then stabilises. For Clustal Omega, default MAFFT and MUSCLE (2 iterations) the SSPA does not stabilise but decreases for large numbers of sequences. In panel S14(c) all sequences that are aligned are also used to predict the secondary structure. This panel presents a convolution of panels S14(a) and S14(b). All aligners benefit from the improved prediction accuracy in panel (a), but Clustal Omega, default MAFFT and 2 iteration MUSCLE less so because of the decrease in alignment accuracy in panel (b). In comparison, panel S14(d) shows the SP score for increasing alignment sizes, which is decreasing for all aligners.

Every program in this study is run using one compute thread only. The reasons for this are the following:

1. Some programs, like ClustalW2, Kalign or Muscle are not parallelised. It is a fairer comparison if parallelised codes are also run in a single-threaded way.
2. It is not obvious, which number of threads, greater than one, to use for parallelised algorithms.
3. Some algorithms, like MAFFT L-INS-i, do not generate reproducible alignments, if run with more than one thread. Pasta and UPP call L-INS-i, and therefore are also affected by this type of irreproducibility.
4. One aspect of this study is that large numbers of alignments have to be generated. According to Amdahl's law, governing the speed-up of parallel algorithms, it is more efficient to execute large numbers of sequential (single threaded) instances in parallel, than to execute parallel instances one after another.

In Figure S15 we plot the SP score against the SSPA score. The input files are sampled only once. The data points appear to be reasonably well correlated, that is, there appears to be a linear correlation between the SP score and the SSPA score. POA, Prank, Dialign and FSA lie below the main block of data; this means that either their respective SSPA scores are higher than would be suggested by the SP scores, or that their SP scores are lower than suggested by their respective SSPA scores. On the other hand, the data point for Praline lies above the main block of data, indicating that its SSPA score is lower than would be suggested by its SP score.

The ordering of the aligners is intuitive: ClustalW2 is one of the less accurate aligners, while MAFFT L-INS-i is one of the higher accuracy aligners. It is also unsurprising, that the average SP score for Prank is uncommonly low – this has frequently been observed for the SP scores of structure based benchmarks. However, Prank's SSPA score falls within the range of other aligners. This suggests, that Prank's gap placement policy does not necessarily frustrate down-stream analysis, like, in this case, secondary structure prediction. It is noteworthy, that Decipher (Wright, 2015), a so far lesser used aligner, is the best aligner for 200 sequences in this study, in terms of both, SSPA and SP scores.

## S7 CHANGE OF SCORES FOR 1000 SEQUENCES

While the correlation of the SP and SSPA scores has been improved, the average SP and SSPA scores themselves did not change appreciably; this can be seen in Supplemental Figures S16 and S17.

For Clustal Omega default the average SP score drops by 2.2%, for Clustal Omega full distance matrix by 2.3%, for Mafft default by 1.4% and for Mafft L-INS-i by 1.6% when going from the old QuanTest data set to the new QuanTest2 data set.

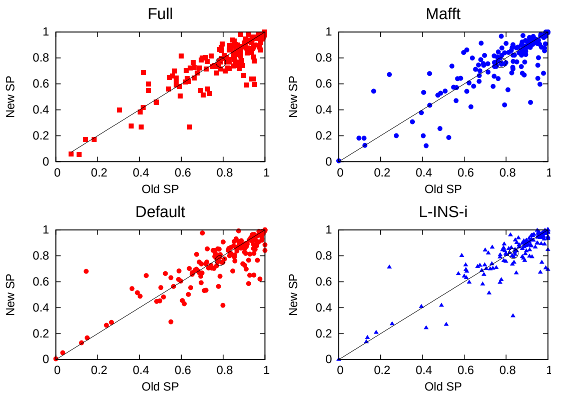


**Fig. S16.** Change in average SP score for Clustal Omega full distance matrix (top-left), Clustal Omega default (bottom-left), Mafft default (top-right) and Mafft L-INS-i (bottom-right). Coloured dots represent individual families, black circle represents average score. Points above the bisectrix indicate higher SP score in the new data selection, points below the bisectrix higher SP score in the old data selection. Scores are averaged over 101 combinations of aligners/options and resamples; alignments are comprised of 1,000 sequences.

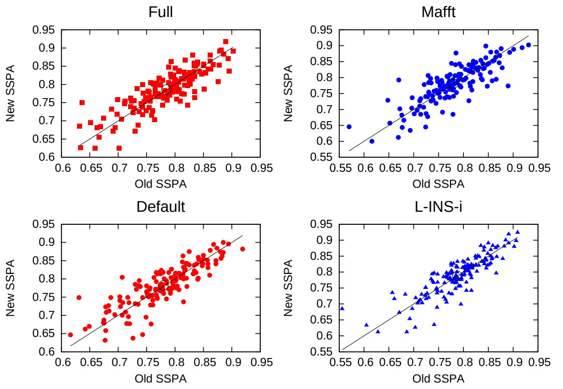


**Fig. S17.** Change in average SSPA score for Clustal Omega full distance matrix (top-left), Clustal Omega default (bottom-left), Mafft default (top-right) and Mafft L-INS-i (bottom-right). Coloured dots represent individual families, black circle represents average score. Points above the bisectrix indicate higher SSPA score in the new data selection, points below the bisectrix higher SSPA score in the old data selection. Scores are averaged over 101 combinations of aligners/options and resamples; alignments are comprised of 1,000 sequences.

For Clustal Omega default the average SSPA score increases by 0.3%, for Clustal Omega full distance matrix by 0.1%, for Mafft default by 0.01% and for Mafft L-INS-i by 0.1% when going from the old QuanTest data set to the new QuanTest2 data set.

## S8 INSTABILITY

It was shown by (Boyce *et al.*, 2015b), that the ordering of the sequences in the input file affects the shape of the guide-tree and therefore the final alignment. We use this instability to our advantage. Simply by re-shuffling the sequences we can increase the number of alignments without having to change the content of the data-files, the alignment software or its command-lines. In Figure S18 we plot the different final alignment lengths if the sequence files are re-shuffled ten times. We plot the absolute alignment lengths as error-bars and the relative variation as bullets. In general, Clustal Omega and MUSCLE produce shorter alignments than MAFFT, especially MAFFT PartTree.

In Figure S19 we plot the instability of the SP scores as the sequences are re-shuffled. We only investigated instability for Clustal Omega, default MAFFT, MAFFT PartTree and MUSCLE with two iterations because these programs are sufficiently fast.

**Table S7.** Pearson Correlation and Spearman Rank Correlation of SP and SSPA scores for 1,800 alignments of new data selection for twelve example families

| Pearson | Spearman | Family |
|---|---|---|
| 0.780026 | 0.696062 | ./ace |
| 0.534204 | 0.531533 | ./Adenylsucc_synt |
| 0.394025 | 0.393699 | ./Asp_Glu_race_D |
| 0.267559 | 0.264153 | ./CH |
| 0.628374 | 0.624518 | ./COX3 |
| 0.539049 | 0.509788 | ./Cu_nir |
| 0.818199 | 0.811538 | ./ghf5 |
| 0.317185 | 0.366256 | ./hla |
| 0.285426 | 0.272449 | ./lacI |
| 0.535267 | 0.436754 | ./rep |
| 0.563972 | 0.532488 | ./response_reg |
| 0.138411 | 0.147114 | ./zf-CCHH |

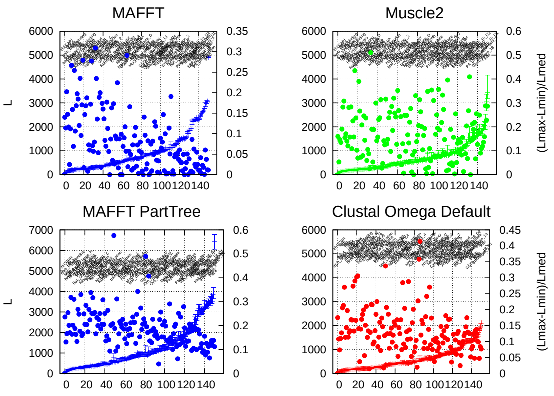


**Fig. S18.** Length instability for different aligners. Results for ten reshufflings of the QuanTest2 families. Data points are length sorted along the x-axis, family names are superimposed at top of each panel. Sorting different for each panel. Error bars give shortest, median and longest alignment length. Length scale on the left. Bullets give relative length variation as difference of longest and shortest length in terms of median length. Variation scale on the right.

FAMSA, which is the fastest algorithm considered here, does not exhibit any alignment instability; therefore we do not re-shuffle sequence data for FAMSA.

## S9 EFFECTIVE TREE SIZES FOR NEW DATA SET

In Figure S6 we showed the distributions of effective guide-tree sizes for the original data selection (Le *et al.*, 2017) for 14 aligners/options in Sievers & Higgins (2018). After having devised a new sequence selection procedure we now show the distribution of effective tree sizes for the new data set in Figure S20.

The effective guide-tree size distributions are based on the alignments, used to generate Figure 4 and Figure 5 in the main paper. While there were 101 alignments created, we could only capture guide-trees for 86 alignments. These were ClustalW2 (5), Default (5), Famsa (1), Full (5), HMM (5), Iter1 (5), Iter2 (5), Kalign (5), Linsi (5), Mafft (5), Muscle (5), Muscle2 (5), Opal (3), PartTree (5), PartTreeDP (5), Pasta (2), PastaM (2), PastaMM (2), PastaMMM (2), Prank (2), TCoffee (2), Viterbi (5). We did not capture guide-trees for Decipher (5), FSA (1), MSAProbs (1), POA (5), Probcons (1), UPP (2). The distribution for the old data selection in Figure S6 was based on 14 alignments.

11 families have median effective tree sizes of less than 400 taxa (down from 40), 41 (=11+30) families have medians of less than 700 taxa (down from 74) and 81 (41+40) families have medians of less than 900 taxa (down from 98). There are no families where all effective trees have more than 900 taxa, however, the trees were generated using more and different aligners/options to the ones in Figure S6.

The last candlestick, labeled 'Summ', gives the distribution for all trees for all families. The overall median effective trees size is 881 taxa (up from 776).

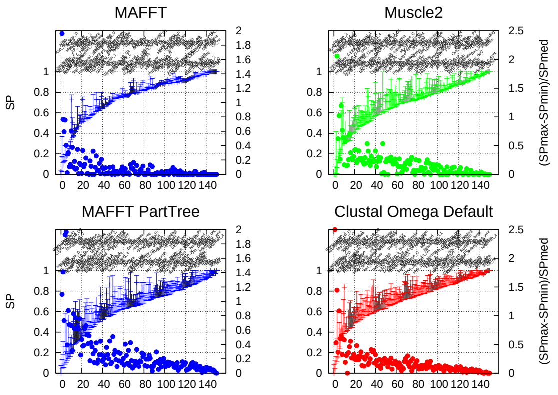


**Fig. S19.** Sum-of-Pairs instability for different aligners. Results for ten reshufflings of the QuanTest2 families. Data points are SP sorted along the x-axis, family names are superimposed at top of each panel. Sorting different for each panel. Error bars give highest, median and lowest SP score. SP scale on the left. Bullets give relative SP variation as difference of highest and lowest SP score in terms of median SP score. Variation scale on the right.

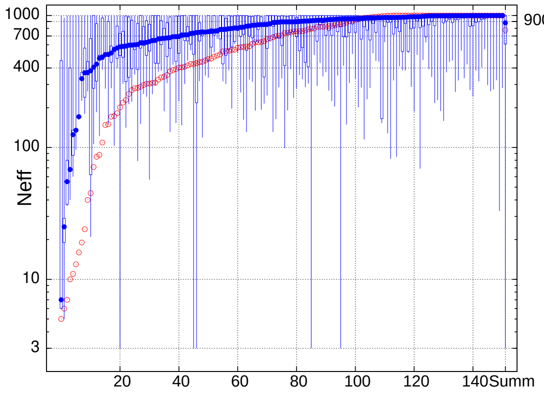


**Fig. S20.** In blue effective tree size distribution for 151 families in Figure 4 in the main paper. Top/bottom quartiles as whiskers, middle quartiles as box, median as bullet. Candlesticks are sorted according to median. Last candlestick (Summ) is distribution for all effective trees. Superimposed in red are the sorted medians of the effective tree sizes for the old data selection. Ordering of families represented by blue and red bullets is not the same, ordering according to median effective tree size only.

This is visualised in Figure S20 by the string of bullets, representing the median effective guide-tree sizes. The values for the old data selection are given as red bullets, the medians for the new selection by blue bullets. There are more guide-trees with larger effective guide-tree sizes with the new data selection than for the old data selection. This is another indicator, that the new data selection in QuanTest2 is better than in QuanTest, as it spreads the reference sequences better amongst the non-reference sequences.

In Table S8 the effective tree size distribution in Figure S20 is shown and compared to the effective size distribution of the artificial guide-trees for the twelve example families in Figure 2 in the main paper (S9).

**Table S8.** Quartiles of the effective tree sizes for the real trees of the new data selection for 1,000 sequences. Q0 indicates the smallest, Q2 the median and Q4 the largest effective tree size.

| Q0 | Q1 | Q2 | Q3 | Q4 | family |
|---|---|---|---|---|---|
| 322 | 724 | 967 | 1000 | 1000 | ace |
| 511 | 864 | 947 | 1000 | 1000 | Adenylsucc_synt |
| 193 | 838 | 853 | 979 | 1000 | Asp_Glu_race_D |
| 524 | 711 | 761 | 1000 | 1000 | CH |
| 128 | 889 | 965 | 1000 | 1000 | COX3 |
| 3 | 497 | 580 | 732 | 1000 | Cu_nir |
| 417 | 691 | 944 | 996 | 1000 | ghf5 |
| 665 | 744 | 763 | 797 | 997 | hla |
| 237 | 754 | 896 | 1000 | 1000 | lacI |
| 153 | 449 | 692 | 991 | 1000 | rep |
| 314 | 678 | 844 | 984 | 1000 | response_reg |
| 305 | 408 | 909 | 997 | 1000 | zf-CCHH |

**Table S9.** Quartiles of the effective tree sizes for the artificial trees of the new data selection for 1,000 sequences. Q0 indicates the smallest, Q2 the median and Q4 the largest effective tree size.

| Q0 | Q1 | Q2 | Q3 | Q4 | family |
|---|---|---|---|---|---|
| 920 | 942 | 976 | 986 | 1000 | ace |
| 902 | 922 | 958 | 991 | 1000 | Adenylsucc_synt |
| 904 | 942 | 963 | 994 | 1000 | Asp_Glu_race_D |
| 903 | 971 | 985 | 1000 | 1000 | CH |
| 900 | 947 | 977 | 989 | 1000 | COX3 |
| 682 | 953 | 977 | 997 | 1000 | Cu_nir |
| 908 | 942 | 968 | 992 | 1000 | ghf5 |
| 813 | 954 | 979 | 994 | 1000 | hla |
| 902 | 946 | 981 | 997 | 1000 | lacI |
| 909 | 955 | 987 | 1000 | 1000 | rep |
| 914 | 970 | 990 | 1000 | 1000 | response_reg |
| 387 | 960 | 985 | 1000 | 1000 | zf-CCHH |

## S10 OTHER BENCHMARKS

In Figure S22 we compare the SSPA scores, obtained for QuanTest2 with scores for other benchmarking methodologies. The top/left panel shows results for the SSPA score against the SP score of the QuanTest data. Points in this panel are the same as in Figure 5 of the main paper.

The top/right panel contrasts the SSPA score with the (SP) Baliscore for BAliBASE3 (Thompson *et al.*, 2005). BAliBASE3 is a collection of 218 alignments, with sizes ranging from 4 sequences to 142 sequences, with a median of 20 sequences. All sequences in the alignment are reference sequences, that is, they contribute to the overall Baliscore. BAliBASE3 attempts to cover different situation, that are encountered when aligning multiple sequences. These scenarios are comprised of (1) alignments of varying degree of similarity and length, (2) alignments that contain orphan sequences, (3) alignments that can be clustered into sub-families, (4) alignments with extensions and (5) alignments that contain insertions. It should be noted, that the Baliscore for the Clustal Omega alignment, using an external HMM, is significantly higher than for all the other aligners/options. The reason for this is that the external HMM was built from the (known) reference alignment. This is a clear case of over-fitting. It was included here nevertheless because (i) there are no ready-made HMMs available for BAliBASE3 and (ii) to demonstrate the potential of external profile alignment. Disregarding this option, the two aligners with the highest average Baliscores are TCoffee and Opal, followed by Clustal Omega's iteration options. Prank and ClustalW2 attain the lowest Baliscores. Pearson correlation for the two scores is 0.675 and Spearman rank correlation is 0.695 (excluding the HMM point).

The bottom/left panel of Figure S22 compares the SSPA score to the PREFAB score (Edgar, 2004). PREFAB is a collection of 1682 protein families of at most 50 sequences. More than three quarter of the families (1293) contain exactly 50 sequences, so

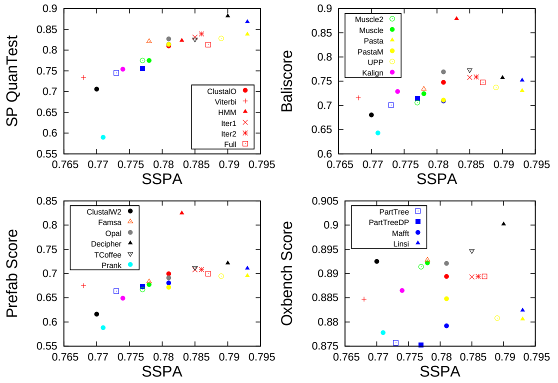


**Fig. S22.** Comparison of SSPA score to different other benchmark scores for various aligners. Top/left panel SSPA score versus SP score of QuanTest data. Top/right panel SSPA score against Baliscore (BAliBASE3). Bottom/left panel SSPA score against Prefab score. Bottom/right panel SSPA score against Oxbench score. Aligner key broken up over four panels, symbol colour and shape same as in Figure S15.

that the median is 50; around 3% of families contain fewer than 10 sequences, around 5% contain fewer than 20 sequences, and around 10% of families contain fewer than 30 sequences. The mean number of sequences is 45.2. The number of reference sequences, whose alignment can be scored, is two for every family. Again, the elevated score for Clustal Omega's HMM option is due to over-fitting. Except for this, the highest performing aligners, in terms of SP score, are Decipher, TCoffee, MAFFT L-INS-i and Clustal Omega's iteration options. Again, Prank and ClustalW2 are the lowest performing aligners in terms of SP score. The Pearson correlation for the QuanTest2 SSPA score and the PREFAB SP score is 0.774, the Spearman rank correlation is 0.849 (excluding the HMM data point).

The bottom/right panel of Figure S22 compares the different aligners in terms of SSPA score and the SP score on the Oxbench data set (Raghava *et al.*, 2003). Oxbench is comprised of 672 protein families. Just over 40% of the families contain two sequences, just under 20% of the families contain three sequences and 10% of the families contain eight or more sequences. The largest family contains 122 sequences. The average number of sequences is 5.7. In Oxbench each sequence is a reference and contributes to the overall score of the alignment. The algorithms with the highest Oxbench score are Decipher, TCoffee, Famsa and ClustalW2. The lowest scores are attained by both MAFFT PartTree options and Prank. It should be noted that MAFFT PartTree is designed to deal with overwhelmingly large numbers of sequences and not small data sets like in Oxbench. The Pearson correlation between SSPA and Oxbench SP score is 0.156 and the Spearman rank correlation is 0.124. The Oxbench ranking of aligners/command-lines is the one that correlates least with the QuanTest2 SSPA score.

## S11 TM-ALIGN

One family (COX3) out of the 151 QuanTest2 families is membrane bound all alpha. Methods that try to improve the alignment of membrane proteins by special treatment are, for example,

PSI/TM-Coffee (http://tcoffee.crg.cat/apps/tcoffee/do:tmcoffee),

Praline (http://www.ibi.vu.nl/programs/pralinewww/) or

TM-Align (http://lms.snu.edu.in/TM-Aligner/index.php).

We could not use Praline, as the web server only accepts sequence files with up to 500 sequences. PSI/TM-Coffee did not successfully complete the alignments. We ran TM-Align interactively for the twelve example families. Its average performance (for the 12 example families) is compared to the other aligners in Table S10.

**Table S10.** SSPA scores for aligners, averaged over 12 example families. TM-Align highlighted in **bold**.

| SSPA | Aligner | SSPA | Aligner |
|---|---|---|---|
| 81.670 | Linsi | 80.499 | PastaM |
| 81.578 | Decipher | 80.475 | TCoffee |
| 81.282 | HMM | 80.233 | Famsa |
| 81.075 | Iter1 | 80.207 | POA |
| 80.865 | Full | 80.169 | Muscle |
| 80.838 | Iter2 | 80.142 | Probcons |
| 80.800 | Muscle2 | 80.047 | Opal |
| 80.754 | PastaMM | 80.042 | PastaMMM |
| 80.675 | UPP | 79.907 | Kalign |
| 80.663 | Pasta | 79.763 | PartTree |
| 80.663 | ClustalW2 | 79.477 | PartTreeDP |
| 80.603 | Mafft | 79.297 | **TM-Align** |
| 80.592 | FSA | 79.275 | Viterbi |
| 80.567 | MSAProbs | 78.900 | Prank |
| 80.542 | Default | | |

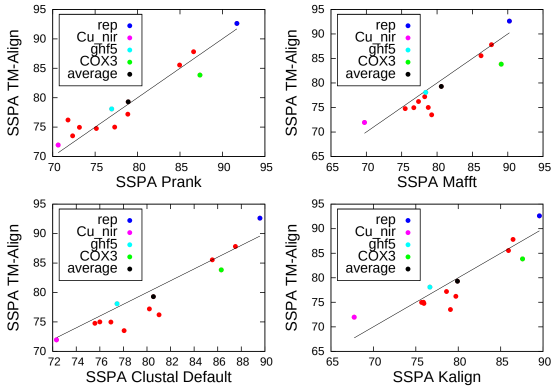


**Fig. S23.** Comparison of SSPA scores for twelve example families for TM-Align versus four other aligners: Prank top-left, Clustal Omega bottom-left, Mafft default top-right, Kalign bottom-right. rep family in blue, Cu_nir in purple, ghf5 in light blue, COX3 in green, other families in red. Average of all 12 families in black. Above the bisectrix TM-Align scores are higher, below the bisectrix TM-Align scores are lower than other aligner.

TM-Align was specifically designed to align membrane bound proteins. However, in none of the 28 comparisons was the SSPA score for COX3 (green) improved (only shown for four of the 28 families in Figure S23). For some aligners certain families did attain higher SSPA score, when compared to the other aligners. Families that were most frequnetly, but not always, improved were the three families high-lighted in Figure S23: rep (blue), Cu_nir (purple) and ghf5 (light blue)

## S12 ADDED SEQUENCES

We want to explore what the effect on the SSPA score would be if unaligned test sequences are aligned onto the reference alignment. We use four different aligners/command-lines:

Clustal Omega default:

```
clustalo --threads=1 --profile1=${REF70}
-i ${eiv}-o ${aln}
```

Clustal Omega full distance matrix:

```
clustalo --threads=1 --profile1=${REF70}
-i ${eiv}-o ${aln}--full
```

Mafft default:

```
mafft --anysymbol --quiet --thread 1
--add ${eiv}${REF70}> ${aln}
```

Mafft L-INS-i:

```
mafft --localpair --anysymbol --quiet
--thread 1 --add ${eiv}${REF70}> ${aln}
```

Here `REF70` is the reference alignment and `eiv` is the full unaligned sequence set, with the three reference sequences removed.

Clustal Omega aligns unaligned sequences onto an aligned profile, which is not to be changed, in the following way. The profile is converted into a HMM. The unaligned sequences are then pre-aligned onto the HMM, and a normal MSA of the previously unaligned sequences is performed. This produces a second profile. In a final step the reference alignment is aligned with the newly generated profile of test sequences.

The reference alignment has by definition an SP score of 1. We observe, however, that the SSPA scores for individual families can be improved with respect to the original alignment (above the bisectrix) or deteriorated (below the bisectrix). In Figure S24 we see that on average (black data point) the effect of adding unaligned sequences onto the reference alignment is marginally positive for Clustal Omega full distance matrix but negative for Clustal Omega default, Mafft default and Mafft L-INS-i.

## S13 DECIPHER DRIVER SCRIPT

Decipher (Wright, 2015) is a software package, written in the statistical computing language R. In this study it is driven by the following R script:

```
library(DECIPHER)
fas <- "vie"
seqs <- readAAStringSet(fas)
aligned <- AlignSeqs(seqs)
writeXStringSet(aligned, "aln")
```

where `vie` and `aln` are the unaligned input and aligned output files, respectively. The above script is invoked as

```
R < rrr --no-save
```

where `rrr` is the name of the above script.

## S14 SSPA SCORING FRAMEWORK

QuanTest2 is comprised of 151 protein families, each containing 1,000 unaligned sequences. For three of these sequences a reliable secondary structure is known; these are the reference sequences. The quality of an alignment of the 1,000 sequences is quantified by predicting secondary structures for the three reference sequences and comparing the predictions to the observed structures. The minimum QuanTest2 benchmark framework therefore is comprised of

- Unaligned test data in the ./Test directory
- Secondary structure data in the ./SS directory
- An evaluation script `./quantest2.py`

In addition there are the following auxiliary directories

- HOMSTRAD Reference alignments (Mizuguchi *et al.*, 1998) in the ./Ref directory
- Pfam names of test sequences and sequence statistics in the ./Aux directory

Alignments should be constructed from the unaligned sequences in the ./Test directory, using the alignment software to be benchmarked. The order of the sequences can be shuffled, however, the labeling of the sequences, especially the three reference sequences, named seq0001, seq0002 and seq0003, should be preserved. The number of sequences to be aligned can be reduced, however, reference sequences seq0001, seq0002 and seq0003 must be retained.
The actual secondary structure prediction is performed using Jpred (Drozdetskiy *et al.*, 2015). Jpred can be be used online via
`http://www.compbio.dundee.ac.uk/jpred/` .
However, here we are using the JPred RESTful API (v.1.5). The main client script (`jpredapi`) can be downloaded as part of an archive
www.compbio.dundee.ac.uk/jpred/downloads/jpredapi.tar.gz .
`jpredapi` is a `perl` script. In order to execute QuanTest2, `perl` has to be installed. Jpred limits the number of predictions to 1,000 per day and per user, as identified by the user's email address.

The evaluation script `./quantest2.py` is a script, written in Python 3; `./quantest2.py` will not run under Python 2. `./quantest2.py` can be executed by explicitly calling (the appropriate version of) Python, or by making the script executable and then calling it directly from the command-line. For example:

```
> python3 ./quantest2.py <argument-list>
```

or

```
> chmod +x ./quantest2.py
> ./quantest2.py <argument-list>
```

The script `./quantest2.py` has to be made executable (`chmod +x`) only once.

The script requires one or many alignments in FASTA format. In FASTA format description lines, beginning with a '>' (greater-than sign) give the sequences names, followed by the actual sequence information. If the format of the alignment is not in FASTA format, then `./quantest2.py` will (presumably) not be able to read the alignment correctly. `./quantest2.py` prepares the alignment, so that it can be used by Jpred to predict the secondary structure of one of the reference sequences. This preparation involves (i) placing the reference sequence, that should be predicted, at the head of the file and (ii) removing all columns in the alignment, where this reference sequence has a gap. (iii) sequences, which after step (ii) consist of gaps only, will be removed. `./quantest2.py` then submits the modified alignment file via `jpredapi`. This is repeated for all three reference sequences, contained in the alignment.

`./quantest2.py` can be invoked for one single alignment or more than one alignment, where the alignment file names possibly can contain wildcards. If more than one family should be evaluated, then `./quantest2.py` requires a list of alignments, followed by a list of structures. There has to be the same number of alignments as there are structure files. The order of the alignment files has to be the same as the order of the structure files.
For example, to determine the SSPA score for the alignment of the AAA family, which is, for example, to be found in the ./Alignment directory, while the structure file is to be found in the ./SS directory, type

```
> ./quantest2.py ./Alignment/AAA.aln
  ./SS/AAA.ss your@email.address.org
```

This will create a log-file, called quantest2.log, containing the predicted states and the SSPA for each of the three reference sequences, as well as the average SSPA for the AAA family. The average score is also printed to screen.

If the SSPA score for the AAA and the aabp families (located in the same directories as above) should be determined, then type

```
> ./quantest2.py ./Alignment/AAA.aln
  ./Alignment/aabp.aln ./SS/AAA.ss
   ./SS/aabp.ss your@email.address.org
```

or

```
> ./quantest2.py ./Alignment/aabp.aln
  ./Alignment/AAA.aln ./SS/aabp.ss
   ./SS/AAA.ss your@email.address.org
```

It should be noted, that (i) the alignment files come ahead of the secondary structure files, (ii) there are the same number of alignment as secondary structure files and (iii) AAA and aabp are in the same order in the list of alignments as in the list of secondary structures.
The following submissions will not work:

```
> ./quantest2.py ./SS/AAA.ss ./SS/aabp.ss
  ./Alignment/AAA.aln ./Alignment/aabp.aln
   your@email.address.org
```

as the structure files come ahead of the alignments;

```
> ./quantest2.py ./Alignment/AAA.aln
  ./Alignment/aabp.aln ./SS/AAA.ss
   ./SS/aabp.ss ./SS/ace.ss
   your@email.address.org
```

as there are three structure files but only two alignment files;

```
> ./quantest2.py ./Alignment/AAA.aln
  ./Alignment/aabp.aln ./SS/aabp.ss
   ./SS/AAA.ss your@email.address.org
```

as the order of alignment files and structure files is not the same;

```
> ./quantest2.py ./Alignment/AAA.aln
  ./SS/AAA.ss ./Alignment/aabp.aln
   ./SS/aabp.ss your@email.address.org
```

as alignment and structure files do no occur in blocks but are interleaved.

It would be very tedious to type out the names for all 151 QuanTest2 families – twice. `./quantest2.py` therefore accepts wild-cards. However, different computing shells sort file names differently, depending on capitalisation and non-alphanumeric characters. For example, in the `bash` shell under Ubuntu

```
> ls Alignment/[aA]sp*.aln
```

returns

```
Alignments/asp.aln
Alignments/Asp_Glu_race_D.aln
```

while

```
> ls SS/[aA]sp*.ss
```

returns

```
SS/Asp_Glu_race_D.ss
SS/asp.ss
```

That is, the `asp` and `Asp_Glu_race_D` families are returned in different order, depending on the file extension. If this particular wild-card logic were to be used for `./quantest2.py`, then the execution would fail because alignment and structure files would not be processed in the same order. One possibility is to sort only up to the file extension:

```
> ls Alignment/[aA]sp*.aln | sort -k1,1 -t.
```

and

```
ls SS/[aA]sp*.ss | sort -k1,1 -t.
```

both return `asp` *before* `Asp_Glu_race_D`. A possible invocation of `./quantest2.py` might therefore be

```
> ./quantest2.py
   $(Alignment/*.aln | sort -k1,1 -t.)
     $(SS/*.ss | sort -k1,1 -t.)
       your@email.address.org
```

Alternatively, one could assign the alignment and the structure files to `bash` variables, making sure that the file names are in the same (consistent) order. One, out of many different, working solutions is

```
> s=$(ls -1 SS/*.ss)
> a=${s//SS\//.\/Alignment}
> b=${a//.ss/.aln}
> ./quantest2.py $b $s
  your@email.address.org
```

## REFERENCES


Bawono,P., Heringa,J., (2014) PRALINE: A Versatile Multiple Sequence Alignment Toolkit, *Methods in Molecular Biology* **1079** pp245-262

Blackshields,G., Sievers,F., Shi,W., Wilm,A., Higgins,D.,G., (2010) Sequence embedding for fast construction of guide trees for multiple sequence alignment, *Algorithms Mol Biol* **14**;5:21

Boyce,K., Sievers,F., Higgins,D.,G., (2015) Reply to Tan et al.: Differences between real and simulated proteins in multiple sequence alignments, *PNAS* **112**(2) E101–E101

Boyce,K., Sievers,F., Higgins,D.,G., (2015) Instability in progressive multiple sequence alignment algorithms, *Algorithms for Molecular Biology* **10**:26

Bradley,R.,K., Roberts,A., Smoot,M., Juvekar,S., Do,J., Dewey,C., Holmes,I., Pachter,L., (2009) Fast Statistical Alignment, *PLOS Computational Biology* **5**(5)

Dalquen,D.A., Anisimova,M., Gonnet,G.H., Dessimoz,C (2012) ALF-A simulation framework for genome evolution, *Mol Biol Evol* **29**(4):1115-1123

Deorowicz,S., Debudaj-Grabysz,A., Gudys,A., (2016) FAMSA: Fast and accurate multiple sequence alignment of huge protein families, *SCIENTIFIC REPORTS* **6**(33964)

Drozdetskiy,A., Cole,C., Procter.,J., Barton,G.,J., (2015) JPred4: a protein secondary structure prediction server, *Nucleic Acids Res* **43**(W1) W389-94

Edgar,R.C., (2004) MUSCLE: multiple sequence alignment with high accuracy and high throughput, *Nucleic Acids Res* **32**(5) 1792-7

Felsenstein,J., (1989) PHYLIP - Phylogeny Inference Package (Version 3.2). *Cladistics* **5**:164-166

Finn,R.D., Bateman,A., Clements,J., Coggill,P., Eberhardt,R.Y., Eddy,S.R., Heger,A.,Hetherington,K., Holm,L., Mistry,J., Sonnhammer,E.L., Tate,J.,. . . Punta,M. (2013). Pfam: the protein families database, *Nucleic acids research* **42**(Database issue) D222-30

Fox,G., Sievers,F., Higgins,D.G., (2015) Using de novo protein structure predictions to measure the quality of very large multiple sequence alignments, *Bioinformatics* **32**(6) 814-820

Higgins,D.,G., Bleasby,A.,J., Fuchs.,R, (1992) CLUSTAL V: Improved software for multiple sequence alignment, *Comput Appl Biosci* **8**(2) 189-191

Katoh,K., Standley,D.M., (2013) MAFFT multiple sequence alignment software version 7: improvements in performance and usability, *Mol Biol Evol* **30**(4) 772-80

Larkin,M.A., Blackshields,G., Brown,N.P., Chenna,R., McGettigan,P.A., McWilliam,H., Valentin,F., Wallace,I.M., Wilm,A., Lopez,R., Thompson,J.D., Gibson,T.J., Higgins,D.G., (2007) Clustal W and Clustal X version 2.0, *Bioinformatics* **23**(21) 2947-8

Lassmann,T., Sonnhammer,E.,L.,L., (2005) Kalign - an accurate and fast multiple sequence alignment algorithm, *BMC Bioinformatics* **6**(298)

Le,Q., Sievers,F., Higgins,D.G., (2017) Protein multiple sequence alignment benchmarking through secondary structure prediction, *Bioinformatics* **33**(9) 1331-1337

Lee,C., Grasso,C., Sharlow,M.,F., (2002) Multiple sequence alignment using partial order graphs, *Bioinformatics* **18**(3) pp452-464

Liu,Y., Schmidt,B., (2014) Multiple Protein Sequence Alignment with MSAProbs, *Methods in Molecular Biology* **1079** pp211-218

Loytynoja,A., (2014) Phylogeny-aware alignment with PRANK, *Methods in Molecular Biology* **1079** pp155-170

Magis,C., Taly,J.,F., Bussotti,G., Chang,J.,M., DiTommaso,P., Erb,I., Espinosa-Carrasco,J., Notredame,C., (2014) T-Coffee: Tree-Based Consistency Objective Function for Alignment Evaluation, *Methods in Molecular Biology* **1079** pp117-129

Mirarab,S., Nguyen,N., Guo,S., Wang,L.,S., Kim,J., Warnow,T., (2015) PASTA: Ultra-Large Multiple Sequence Alignment for Nucleotide and Amino-Acid Sequences, *Journal of Computational Biology* **22**(5) pp377-386

Mizuguchi,K., Deane,C.,M., Blundell,T.,L., Overington,J.,P., (1998) HOMSTRAD: a database of protein structure alignments for homologous families, *Protein Sci* **7**(11) 2469-71

Morgenstern,B., (2014) Multiple Sequence Alignment with DIALIGN, *Methods in Molecular Biology* **1079** pp191-202

Nguyen,N.,P.,D., Mirarab,S., Kumar,K., Warnow,T., Ultra-large alignments using phylogeny-aware profiles, *Genome Biology* **16**(124)

Pavlopoulos,G.A., Soldatos,T.G., Barbosa-Silva,A., Schneider,R (2010) A reference guide for tree analysis and visualization, *BioData Min* **3**(1)

Price,M.N,. Dehal,P.S., Arkin,A.P., (2010) FastTree 2 - Approximately Maximum-Likelihood Trees for Large Alignments, *PLoS ONE* **5**(3) e9490

Raghava,G.,P.,S., Searle,S.,M.,J., Audley,P.,C., Barber,J.,D., Barton,G.,J., (2003) OXBench: A benchmark for evaluation of protein multiple sequence alignment accuracy, *BMC Bioinformatics* **34**:47

Roshan,U., (2014) Multiple Sequence Alignment Using Probcons and Probalign, *Methods in Molecular Biology* **1079** pp147-153

Sievers,F., Wilm,A., Dineen,D.G., Gibson,T.J., Karplus,K., Li,W., Lopez,R., McWilliam,H., Remmert,M., Söding,J., Thompson,J.D., Higgins,D.G., (2011) Fast, scalable generation of high-quality protein multiple sequence alignments using Clustal Omega, *Mol Syst Biol* **7** 539

Sievers,F., Dineen,D., Wilm,A., Higgins,D.G., (2013) Making automated multiple alignments of very large numbers of protein sequences, *Bioinformatics* **29**(8), 989-995

Sievers,F., Hughes,G.,M., Higgins,D.G., (2014) Systematic Exploration of Guide-Tree Topology Effects for Small Protein Alignments, *BMC Bioinformatics* **15** 338

Sievers,F., Higgins,D.,G., (2018) Clustal Omega for making accurate alignments of many protein sequences, *Tools for Protein Science* **27**(1) 135-145

Thompson,J.D., Koehl,P., Ripp,R., Poch,O. (2005) BAliBASE 3.0: latest developments of the multiple sequence alignment benchmark, *Proteins*, **61**(1):127-36

Wheeler, T.J. and Kececioglu, J.D., (2007) Multiple alignment by aligning alignments, *Bioinformatics* **23**(13) i559-i568

Williams,T.,C., Kelley,H.,B., Bröker,J., Campbell,R., Cunningham,D., Denholm,E., Elber,R., Fearick,C., Grammes, L., Hart.L., (2011) Gnuplot 4.5: An Interactive Plotting Program, http://www.gnuplot.info

Wright,E.,S., (2015) DECIPHER: harnessing local sequence context to improve protein multiple sequence alignment, *BMC Bioinformatics* **16**(322)

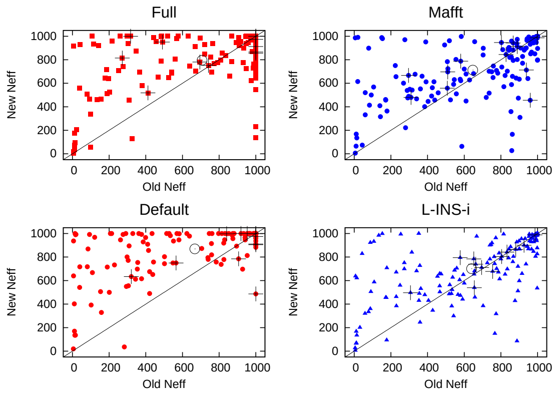


**Fig. S21.** Comparison of effective guide-tree sizes for old and new data selection for Clustal Omega full distance matrix (top-left), Clustal Omega default (bottom-left), Mafft default (top-right) and Mafft L-INS-i (bottom-right). Coloured dots represent individual families, black circle represents average score. Points above the bisectrix indicate higher effective guide-tree size in the new data selection, points below the bisectrix higher effective guide-tree size in the old data selection. Values are averaged over 101 combinations of aligners/options and resamples; alignments are comprised of 1,000 sequences.

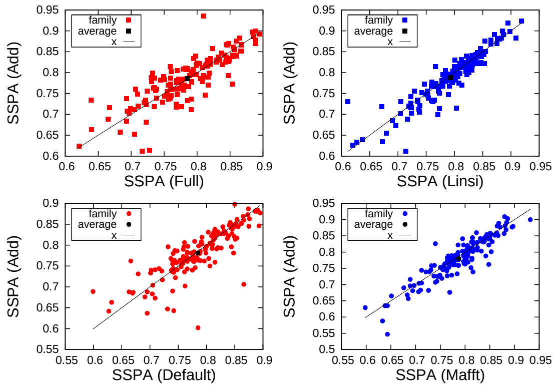


**Fig. S24.** Comparison of SSPA scores for four aligners when non-reference sequences are added to the reference alignments against scores when all 1,000 sequences are aligned from scratch. Clustal Omega full distance matrix top-left, Clustal Omega default bottom-left, Mafft L-INS-i top-right, Mafft default bottom-right. Results for individual families in red/blue, average scores in black. Points below the bisectrix have a higher SSPA score in the original scheme, points above the bisectrix have a higher score, when unaligned sequences are added to reference alignment.
